# A candidate loss-of-function variant in SGIP1 causes synaptic dysfunction and recessive parkinsonism

**DOI:** 10.1016/j.xcrm.2024.101749

**Published:** 2024-09-26

**Authors:** Marianna Decet, Patrick Scott, Sabine Kuenen, Douja Meftah, Jef Swerts, Carles Calatayud, Sandra F. Gallego, Natalie Kaempf, Eliana Nachman, Roman Praschberger, Nils Schoovaerts, Chris C. Tang, David Eidelberg, Samir Al Adawi, Abdullah Al Asmi, Ramachandiran Nandhagopal, Patrik Verstreken

**Affiliations:** 1 VIB-KU Leuven Center for Brain & Disease Research, 3000 Leuven, Belgium; 2 KU Leuven, Department of Neurosciences, Leuven Brain Institute, 3000 Leuven, Belgium; 3 Laboratory of Molecular Biology, Sainte-Justine University Hospital Center, Montréal QC H3T 1C5, Canada; 4 Laboratory of Pulmonary Physiology, Department of Pediatrics, Sainte-Justine University Hospital Center, Montréal QC H3T 1C5, Canada; 5 Center for Neurosciences, The Feinstein Institutes for Medical Research, Manhasset, NY 11030, USA; 6 Department of Behavioral Medicine, College of Medicine & Health Sciences, Sultan Qaboos University, Al Khod 123, Muscat, Oman; 7 Neurology Unit, Department of Medicine, College of Medicine and Health Sciences, Sultan Qaboos University, Al Khod 123, Muscat, Oman

**Keywords:** young-onset parkinsonism, seizures, SGIP1 variant, synaptic proteostasis, multivesicular body

## Abstract

Synaptic dysfunction is recognized as an early step in the pathophysiology of parkinsonism. Several genetic mutations affecting the integrity of synaptic proteins cause or increase the risk of developing disease. We have identified a candidate causative mutation in synaptic “SH3GL2 Interacting Protein 1” (SGIP1), linked to early-onset parkinsonism in a consanguineous Arab family. Additionally, affected siblings display intellectual, cognitive, and behavioral dysfunction. Metabolic network analysis of [^18^F]-fluorodeoxyglucose positron emission tomography scans shows patterns very similar to those of idiopathic Parkinson’s disease. We show that the identified *SGIP1* mutation causes a loss of protein function, and analyses in newly created *Drosophila* models reveal movement defects, synaptic transmission dysfunction, and neurodegeneration, including dopaminergic synapse loss. Histology and correlative light and electron microscopy reveal the absence of synaptic multivesicular bodies and the accumulation of degradative organelles. This research delineates a putative form of recessive parkinsonism, converging on defective synaptic proteostasis and opening avenues for diagnosis, genetic counseling, and treatment.

## Highlights

• Two Omani siblings present with parkinsonism and harbor a mutation in *SGIP1*
• The mutation in *SGIP1* results in a loss of function
• Loss of *SGIP1* function in *Drosophila* affects synaptic transmission
• *SGIP1* is required for the regulation of degradative organelles at synapses

## Graphical abstract

**Figure:**
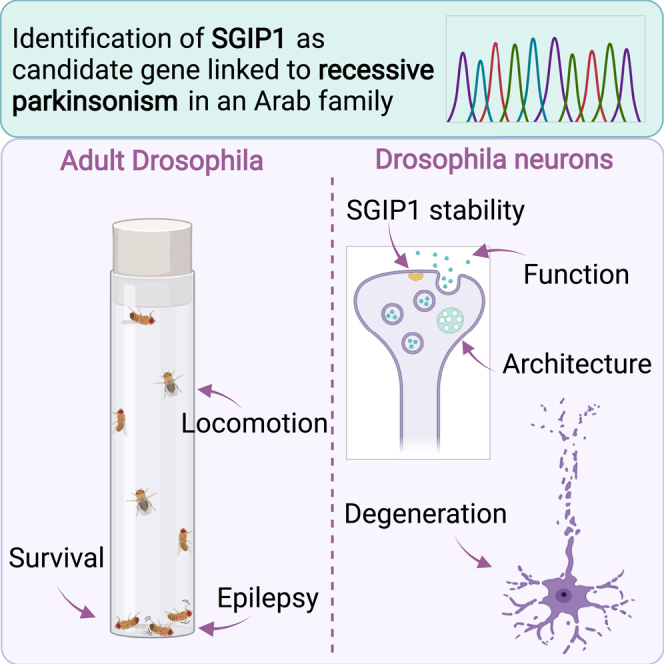

## Teaser

Decet et al. identify a mutation in *SGIP1* putatively linked to early-onset parkinsonism in an Arab family. This mutation results in a loss of function, and *SGIP1* fly mutants suffer from movement defects, synaptic dysfunction, and neurodegeneration. Their findings suggest a potential form of recessive parkinsonism, impacting synaptic proteostasis.

## Introduction

Parkinson’s disease (PD) is phenotypically characterized by bradykinesia, rest tremor, rigidity, postural instability, levodopa responsiveness, medication-related motor complications, non-motor dysfunction, and the loss of dopaminergic neurons in the *substantia nigra*.^1^ This often coincides with the intra-neuronal accumulations of Lewy bodies (LBs). Synaptic dysfunction is emerging as a crucial step in the early phase of disease pathogenesis.^2^ The extensive synaptic connections of the *nigral* neurons in the striatum appear to be particularly susceptible, and their dysfunction and degeneration trigger striatal output imbalance that is involved in the onset of motor symptoms.^3^^,^^4^ Furthermore, considering the concurrent formation of protein aggregates within LB, it has been proposed that a potential model for the development of PD involves the failure of synaptic homeostasis as a crucial molecular and cellular driver.^5^^,^^6^^,^^7^^,^^8^^,^^9^

The monogenic form of parkinsonism (OMIM phenotypic series 168600) is genetically and clinically heterogeneous, including several rare early-onset cases due to recessive mutations in the genes *SH3GL2*, *SYNJ1*, and *DNAJC6*, which encode proteins with critical synaptic functions such as Endophilin A1, Synaptojanin 1 and Auxilin, respectively.^10^^,^^11^^,^^12^ These mutations lead to a spectrum of clinical manifestations that extend beyond the typical motor symptoms of PD and often include intellectual disability and seizures.^10^^,^^12^ The molecular function of EndophilinA1, Synaptojanin1, and Auxilin plays a crucial role in synaptic vesicle (SV) cycling and neurotransmission and influences (synaptic) lipid metabolism.^13^^,^^14^ In addition, our research and that of others have shown their role in controlling synaptic proteostasis by regulating autophagy-lysosomal pathways.^8^^,^^13^^,^^15^ This regulation potentially occurs through the modulation of specific lipid levels that are involved in shaping membranes (curvature) and recruiting specific proteins, such as those involved in autophagy.^8^^,^^13^^,^^16^ Additionally, recent genome-wide association studies (GWASs) have identified genomic loci in the vicinity of genes that encode synaptic proteins, providing further evidence supporting the idea that defective synaptic homeostasis contributes to the disease pathogenesis.^17^^,^^18^ Although these synaptic proteins interact with each other and are regulated by common kinases, including PD-mutated LRRK2 and phosphatases (like Calcineurin), the precise coordination of their functions in synaptic activity and proteostasis, as well as the comprehensive composition of this protein network, is not yet fully understood.

SH3GL2 Interacting Protein 1 (SGIP1) is a brain-specific adaptor protein that was initially identified as an interactor of Endophilins^19^ and is thought to function in SV endocytosis.^20^^,^^21^ We identified an Arab family with an unexplained form of young-onset parkinsonism, and, utilizing direct sequencing, homozygosity mapping, whole-exome sequencing, co-segregation analysis, and functional studies, we report a candidate variant ([GeneBank: NM_032291] c.2080T>G [p.W694G]) in the *SGIP1* gene as the most plausible underlying cause of disease. We show that W694G causes a loss of function, and we created new *Drosophila* models that recapitulate cardinal features of disease, including movement problems, seizures, and neurodegeneration, including dopaminergic synapse loss. These animals also suffer from synaptic defects, including the accumulation of degradative organelles. Hence, loss of *SGIP1* function, similar to pathogenic mutations in other synaptic proteins, causes defects in synaptic proteostasis. Our work not only identifies a plausible disease-causing variant in *SGIP1* but also importantly adds to the role of synaptic proteostasis impairment in the pathogenesis of recessive parkinsonism.

## Results

### Clinical phenotype of subject III:1 (proband) and III:3

**Figure 1:**
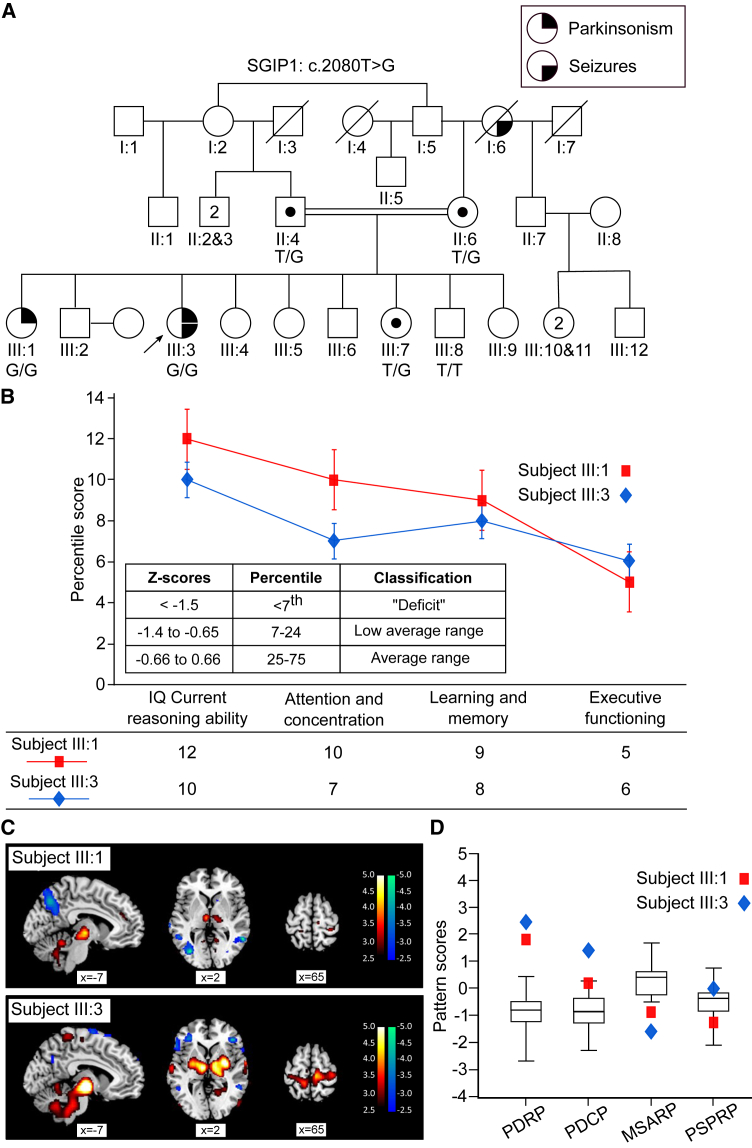
Early-onset parkinsonism manifested in 2 siblings of a consanguineous Arab family (A) Pedigree of the Arab family with 2 affected individuals (subjects III:1 and III:3) manifesting with young-onset parkinsonism with or without seizures and segregating the *SGIP1* variant: NM_032291: c.2080T>G in an autosomal recessive mode of inheritance in the subjects who were sequenced (Figure 2A). The genotype (and carrier status) is mentioned below the tested participants. The index patient is marked with an arrow. (B) Neuropsychological assessment (percentile scores of different cognitive domains) of the affected siblings subjects III:1 (red squares) and III:3 (blue diamonds) with recessive parkinsonism along with intellectual and cognitive dysfunction. Points: mean percentile score ± SD. (C) Single-case voxel-wise analysis of FDG PET scans of the affected individuals subjects III:1 and III:3 with recessive parkinsonism showing abnormally increased (red-yellow, see color scale) and decreased (blue-green, see color scale) regional metabolism in the brain of each patient, compared to an age-matched healthy control (HC) group (*n* = 18). Significant voxels were displayed at a threshold of *p* < 0.01. (D) Network analysis of the FDG PET scans of subjects III:1 (red squares) and III:3 (blue diamonds) showing that both patients exhibited characteristic expression (subject scores) of metabolic patterns for idiopathic Parkinson’s disease (PD), but not multiple system atrophy (MSA) and progressive supranuclear palsy (PSP), compared to the HC group (Box and Whisker plots; *n* = 18). PDRP and PDCP, PD-related motor and cognitive metabolic patterns; MSARP, MSA-related metabolic pattern; PSPRP, PSP-related metabolic pattern. See also Table S1 and Video S1.

We identified two affected sisters (subjects III:1 and III:3), born of consanguineous Arab parents (Figure 1A) who manifested young-onset parkinsonism. The proband of the family, affected female subject III:1, presented with an insidious onset of asymmetrical rest tremor (left more than right hands), progressive bradykinesia, and limb rigidity at the age of 19 years. She had an improvement of her short, shuffling gait with levodopa and pramipexole, in addition to the emergence of off-period foot dystonia, and postural instability (retropulsion and frequent falls). Additional non-motor dysfunction including behavioral, intellectual, and cognitive dysfunction characterized by anger outburst, beating of relatives, verbally abusive utterance, and low performance scores in intellectual and cognitive tasks (Figure 1B, red squares) posed limitations to the dose escalation of dopaminergic medications to control motor symptoms. She did not have *Kayser*-*Fleischer rings*. At age 30, she scored 41 points on the motor component of the Movement Disorders Society Unified Parkinson Disease Rating Scale (MDS-UPDRS Part III). After 11 years of disease progression, there was a significant problem in motor performance (Video S1). The other subject III:3 developed a similar levodopa/dopamine agonist-responsive parkinsonian phenotype of 6-year duration with onset of the disease at age 22 (Video S1). Similarly, to subject III:1, she also presented behavioral, intellectual, and cognitive dysfunction (Figure 1B, blue diamonds) and scored 46 points on the MDS-UPDRS Part III scale. Additionally, from age 10, she suffered generalized tonic clonic seizures that were initially treated with sodium valproate, followed by therapeutic replacement with levetiracetam and lamotrigine for possible side effects such as worsening of parkinsonism and future development of postural tremor and polycystic ovarian disease. The other relatives did not show such problems (Figure 1A). The basic metabolic panel (including their calcium, phosphate, uric acid, and ceruloplasmin and thyroid profile) and cranial MRI scans were unremarkable in both affected subjects. A detailed overview of the genetics and clinical characteristics of both patients is presented in Table S1. Therefore, we clinically diagnosed both affected individuals with early-onset parkinsonism.

Video S1. Clinical video of subject III:1 and III:3 manifesting with young-onset parkinsonism, related to Figure 1 Video illustrating the motor performance of subject III:1 and subject III:3 with young-onset parkinsonism

### FDG PET imaging revealed brain metabolic abnormalities consistent with idiopathic PD

To confirm the clinical diagnosis of PD, we analyzed [^18^F]-fluorodeoxyglucose (FDG) positron emission tomography (PET) scans from both patients and conducted single-case voxel-wise analysis to search for regional metabolic abnormalities in the scan of each patient. Subject III:1 exhibited a significant increase in regional metabolism in the thalamus, pons, and cerebellum, with abnormal reductions in parietal and occipital association cortex (Figure 1C, top). We then determined whether two previously characterized PD-related metabolic patterns, correlating respectively with motor and cognitive symptoms,^22^ were present in these patients. Indeed, the expression of the PD-related motor pattern (PDRP) was elevated in Subject III:1 (score = +1.85; Figure 1D, red squares) compared to 18 age-matched healthy subjects (Figure 1D, boxplot), whereas the expression of the PD-related cognitive pattern (PDCP) was normal in this individual (score = +0.27). Additionally, the finding of greater PDRP expression compared to PDCP (delta = +1.59) in this patient is consistent with an idiopathic PD as opposed to a clinically similar atypical parkinsonian variant, such as multiple system atrophy (MSA) or progressive supranuclear palsy (PSP).^23^ This accords with the low expression of the previously characterized metabolic patterns for these disorders (MSA-related metabolic pattern [MSARP] score = −0.84; PSP-related metabolic pattern [PSPRP] score = −1.67), observed in this individual (Figure 1D, red squares). When subject scores for PDRP, MSARP, and PSPRP from this subject were entered into an automated image-based algorithm for differential diagnosis,^22^^,^^24^ the resulting image-based classification was idiopathic PD with high probability (99.5%) (Table S1).

Subject III:3 exhibited regional metabolic abnormalities similar to those seen in Subject III:1, with increased activity in the putamen, globus pallidus, thalamus, motor cortex, pons, and cerebellum, as well as reduced activity in the frontal and parietal cortex (Figure 1C, bottom). By the same token, expression values for PDRP and PDCP were both elevated (scores = +2.47 and +1.43, respectively; Figure 1D, blue diamonds), which, along with PDRP predominance (delta = +1.04), supports the diagnosis of idiopathic PD. Accordingly, this patient had comparatively low MSARP and PSPRP expression levels (scores = −1.57 and +0.01, respectively; Figure 1D, blue diamonds) and was also classified as an idiopathic PD with high probability (99.3%) by the image-based algorithm (Table S1).

### Molecular genetic testing identified *SGIP1*

**Figure 2:**
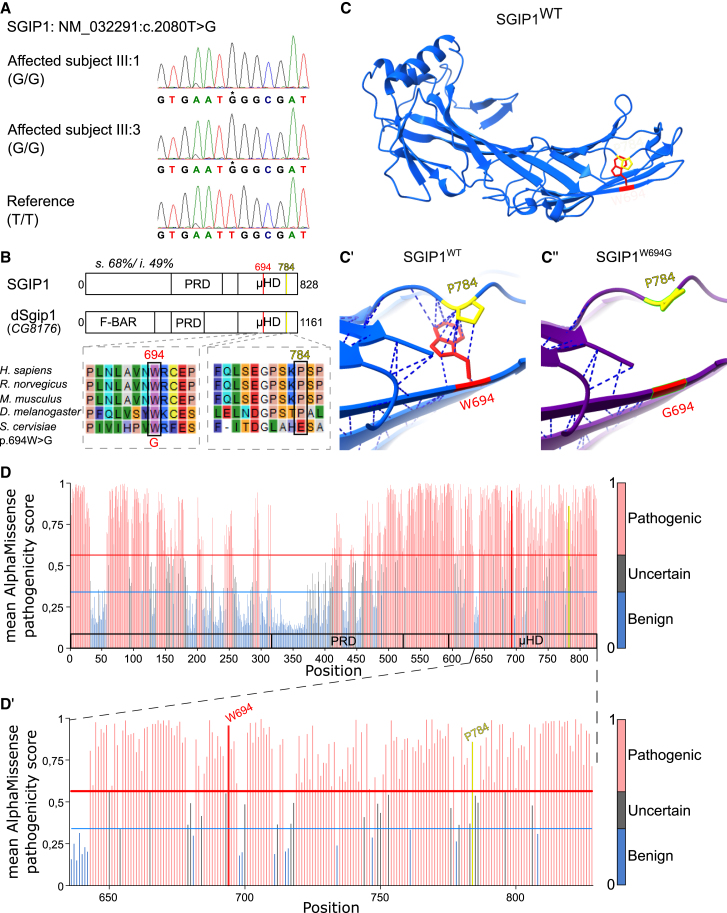
The variant causing early-onset parkinsonism in subjects III:1 and III:3 is located in *SGIP1* and is predicted to be pathogenic (A) Sanger sequencing of the target region within exon 22 of the *SGIP1* gene harboring the c.2080T>G variant. DNA sequencing revealed a homozygous G/G nucleotide change in the *SGIP1* gene (c.2080) in subjects III:1 and III:3 presenting with parkinsonism, compared to a T/T in the reference genome at this position. (B) The protein alignment shows evolutionary conservation of the mutated tryptophan (at position 694 in human SGIP1 (p.W694) and 1003 (p.W1003) in *Drosophila* dSgip1). The μHD of the human and fly proteins share 68% of similar and 49% of identical amino acids. Note that the proline interacting with the mutated W (in C) is also well conserved (at position 784 in human SGIP1 [p.P784] and 1092 [p.P1092] in *Drosophila* dSgip1). (C–C″) AlphaFold-predicted protein structure of the μHD domain of isoform 1 of SGIP1 (AF-Q9BQI5-F1-v4: position 531–828) revealing a loss of hydrophobic contacts (dashed lines) between W694 (*red*) and P784 (*yellow*) when W694 is substituted by a glycine (G694). (D and D′) Quantification of the average AlphaMissense (AM) pathogenicity score for each amino acid for the complete SGIP1 protein sequence (D); enlarged for part of the μHD (D′). Each residue is colored according to the average AM pathogenicity score of each given residue (out of 19 possible amino acid changes per residue). Red, likely pathogenic variants (meaning overall changes of this residue to any other residue are predicted as pathogenic); blue, likely benign variants; gray, ambiguous variants. Note that overall changes at position 694 (marked in red) and 784 (marked in yellow) are likely pathogenic. See also Tables S2 and S3.

To identify genetic mutations in these patients, we first performed targeted sequencing. This revealed no clinically significant DNA variants or copy-number variations in the *PARK2* and *PLA2G6* genes. Furthermore, whole-exome sequencing did not identify pathogenic (or likely pathogenic) variants in the *PINK1*, *SYNJ1*, and *PODXL* genes and other known genes associated with early-onset parkinsonism. Additional homozygosity (runs of homozygosity [ROH]) analyses identified five shared genomic regions totaling 55.4 Mb between the two affected individuals (subjects III:1 and III:3) (Table S2). These genomic positions were analyzed for known OMIM gene entries that list parkinsonism. Within the homozygous region on chromosome 1 (56811604–74107645), *DNAJC6* (*1p31.3*) was reported to be associated with young-onset parkinsonism.^12^^,^^25^^,^^26^ However, direct sequencing of *DNAJC6* in the proband revealed no clinically significant mutations, deletions, or duplications. We then restricted the exome sequencing data analysis to those shared homozygous regions. Within the ROHs, restricting the analysis with compatible expression profile, allele frequency, and protein altering variation predictors, we identified *SGIP1* (GeneBank: NM_032291.4) as the only plausible candidate gene in this family. This gene would be a candidate gene causing parkinsonism as it was not shared in GeneMatcher, nor (yet) listed in the Parkinson’s Disease DNA Variant Browser from the Global Parkinson’s Genetics Program (GP2) dataset.^27^^,^^28^^,^^29^ The affected kindred subjects (subjects III:1 and III:3) carried a homozygous missense variant consisting of a T>G transition at nucleotide position 2080 (c.2080T>G) in exon 22. The unaffected parents (subjects II:4 and II:5) and their unaffected younger sister (subject III:7) were heterozygous carriers of the *SGIP1* variant c.2080T>G (Figure 1A). This variant was absent from the main variation databases, including gnomAD (v4.1.0), Greater Middle East Variome, dbSNP, and ClinVar. The genomic constraint metric (depletion of variant) for the 1 kb region surrounding the observed variant *SGIP1* had a *Z* score of 2.38, representing the top 10% of the constrained non-coding regions.^30^ Sanger sequencing validated the identity of the *SGIP1* variant and its absence in the homozygous state in the unaffected relatives (Figures 1A and 2A).

The c.2080T>G mutation in *SGIP1* causes the substitution of a non-polar aromatic tryptophan to an aliphatic glycine at amino acid position 694 (p.W694G) in the μ-homology domain (μHD) of the protein (Figure 2B). We used AlphaFold^31^ to model the previously crystalized structure of the SGIP1 μHD domain^32^ and found that pathogenic substitution could cause a loss of hydrophobic interactions between W694 and P784 (Figure 2C-2C″). Since both residues are well conserved across species (Figure 2B), mutations in either would likely disrupt protein function. To test this hypothesis *in silico*, we resorted to AlphaMissense, an AI model that predicts the pathogenicity of amino acid substitutions.^33^ This confirmed that W694G is likely pathogenic potentially by destabilizing the protein (Figure 2D and 2D′). Other *in silico* prediction tools agreed that this substitution is deleterious or disease causing (Table S3). Furthermore, P784 substitutions are also predicted to be pathogenic, as are many residues in the μHD domain (Figure 2D-D′), and, while there is a very rare SNP affecting this amino acid reported in gnomAD, none of its carriers are homozygous.

### SGIP1^W694G^ decreased protein stability

**Figure 3:**
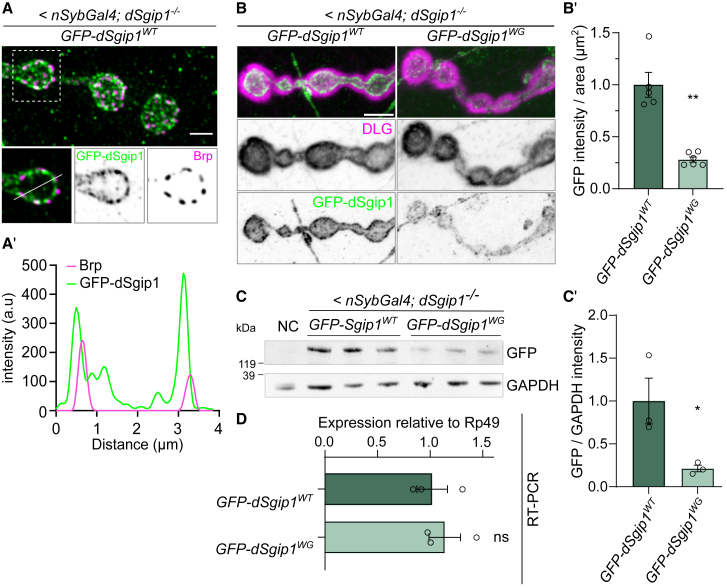
*Drosophila* dSgip1 localizes to synapses and the pathogenic mutant lowers protein stability (A) Maximum projection composite Airyscan confocal image of an NMJ expressing GFP-dSgip1^WT^ (*<nSybGal4; dSgip1*^*−/−*^) and labeled with anti-GFP (green) and anti-Brp (nc82, magenta) antibodies, where Brp marks active zones. Insert: a single confocal section. Scale bar: 2 μm. (A′) Fluorescence intensity plot (arbitrary units) along the line indicated in insert in (A). (B) Representative maximum projection composite confocal images of NMJs of flies expressing wild-type or mutant GFP-dSgip1 (GFP-dSgip1^WT or W694G^) (*<nSybGal4; dSgip1*^*−/−*^) and stained with anti-GFP (green) and anti-DLG antibodies (magenta), where DLG marks the post-synaptic site. Scale bar: 5 μm. (B′) Quantification of the average GFP intensity per NMJ area. 4 NMJs per animal were analyzed, *n* ≥ 5 animals per genotype. Statistical significance: unpaired t test with Welch’s correction. ∗∗*p* < 0.01. Bars: mean ± SEM. (C and C′) Western blot from adult *Drosophila* head lysates of indicated genotypes labeled with anti-GFP marking GFP-dSgip1 and anti-GAPDH (loading control) (C) and the quantification of GFP-dSgip1 protein levels (C′). Values are relative to GAPDH for the three replicates of each genotype. NC (negative control): flies not expressing any GFP construct. Statistical significance: unpaired t test. ∗*p* < 0.05. Bars: mean ± SEM; points are individual values and *n* ≥ 3 per genotype. (D) Quantitative RT-PCR to assess GFP-dSgip1 expression levels in adult head extracts relative to Rp49. RT-PCR primers were designed against dSgip1. While RNA levels of the W694G variant and wild-type variant are indistinguishable, there is less W694G mutant protein at synapses than wild-type protein (C), indicating the mutations destabilize dSgip1. Statistical significance: unpaired t test. ns, not significant. Bars: mean ± SEM; points are individual values and *n* ≥ 3.

Since SGIP1 is well conserved across species (Figure 2B), we assessed whether the W694G mutation destabilized the protein using fruit flies that express the *SGIP1* ortholog, *dSgip1* (*CG8176*). The critical μHD was 68% similar and 49% identical to human counterparts at the amino acid level, and both W694 and P784 were conserved, respectively, corresponding to p.W1003 and p.P1092 in the dSgip1 protein (Figure 2B). We resorted to the UAS-GAL4 system for targeted expression of transgenes and generated transgenic flies that allowed cell-specific expression of GFP-tagged dSgip1^W694G^ (hereafter dSgip1^WG^; mutant protein) and GFP-tagged dSgip1^WT^ (wild-type protein). When crossed to flies expressing GAL4 under a neuronal promoter (<*nSybGal4*), GFP-tagged dSgip1^WG^ and dSgip1^WT^ were expressed in neurons. Imaging the neuromuscular junctions (NMJs) of third-instar *Drosophila* larvae revealed that both the wild-type and the mutant proteins were localized to the presynaptic terminals (Figures 3A and 3B), similar to rodent SGIP1.^34^^,^^35^ Further analysis revealed that the proteins were clustered in “peri-active zones,” areas of the synapse where vesicle endocytosis occurs (Figures 3A and 3A′). Although both the mutant and wild-type proteins were synaptic, the expression level of the mutant protein was ∼30% lower than the levels found in animals expressing the wild-type protein (Figures 3B and 3B′). Similarly, western blot analyses of adult head extracts expressing dSgip1^WG^ or dSgip1^WT^ also showed decreased levels of the mutant protein compared to the wild-type protein (Figures 3C and 3C′), while they expressed equal levels of the mutant or wild-type mRNA (Figure 3D). Hence, dSgip1 localized to peri-active zones at synapses and the W694G mutation in *SGIP1*, identified in the Arab kindred, resulted in reduced SGIP1 protein levels.

### *dSgip1* loss-of-function mutants exhibited increased lethality and behavioral dysfunction

**Figure 4:**
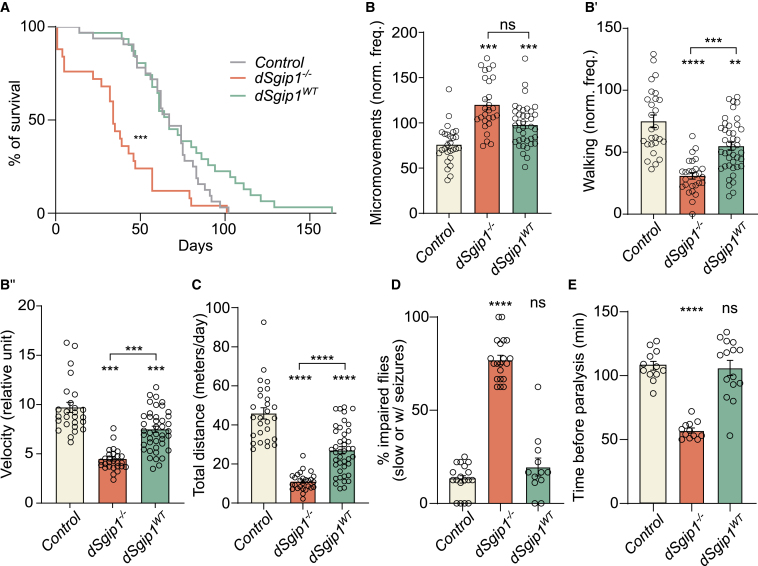
*dSgip1* loss-of-function mutants exhibit increased lethality and severe behavioral dysfunction (A) Survival rate of flies of the indicated genotypes over time. Statistical significance: Mantel-Cox test ∗∗*p* < 0.01. Number of animals ≥ 25 per genotype. (B and B′) Activity monitoring carried out with young (5-day-old) flies of indicated genotypes housed in ethoscopes. Normalized frequency of the indicated behavior: micromovement (B) and walking (B′). Number of animals ≥ 27 per genotype, in two independent experiments. Statistical significance: one-way ANOVA. ∗∗∗*p* < 0.001; ns, not significant, compared to control and *dSgip1*^*WT*^ animals. Bars: mean ± SEM, and points are individual values. (B″) Quantification of the velocity of single flies of indicated genotypes. Number of animals ≥ 27 per genotype, in two independent experiments. Statistical significance: one-way ANOVA. ∗∗∗*p* < 0.001, compared to control and *dSgip1*^*WT*^ animals. Bars: mean ± SEM, and points are individual values. (C) Total distance walked by each fly housed in the ethoscopes of indicated genotypes. Statistical significance: one-way ANOVA. ∗∗∗∗*p* < 0.0001, compared to control and *dSgip1*^*WT*^ animals. Bars: mean ± SEM, and points are individual values. (D) Percentage of young (5-day-old) impaired flies of indicated genotypes. Impaired is uncoordinated or showing seizure-like behavior following 10 s of vortexing. Each data point represents a group of 7–10 flies. Statistical significance: one-way ANOVA. ∗∗∗∗*p* < 0.0001; ns, not significant, compared to control. Bars: mean ± SEM, and points are individual values. (E) Time (min) before each fly of indicated genotypes shows complete paralysis. Flies were exposed to 38°C. Number of tested flies ≥ 12 per genotype. Statistical significance: one-way ANOVA. ∗∗∗∗*p* < 0.0001; ns, not significant, compared to control. Bars: mean ± SEM, and points are individual values. See also Figure S1.

Next, we created fruit flies in which the endogenous *dSgip1* gene was mutated enabling us to analyze the implications of the loss of dSgip1 function. Using CRISPR-Cas9 and a targeting strategy recently described,^36^ we knocked out exon 1 of the *dSgip1* gene (*dSgip1*^*−/−*^) and confirmed by reverse-transcription PCR (RT-PCR) that the expression of *dSgip1* was abolished (Figures S1A and S1B). We also created a wild-type knockin (*dSgip1*^*WT*^) using a knockin strategy^36^ to serve as an additional control and show that *dSgip1* expression is restored in these knockin animals (Figures S1A and S1B). To study the effect of loss of *dSgip1* on longevity, we first monitored their lifespan and found that *dSgip1*^*−/−*^ mutants lived significantly shorter than wild-type controls and *dSgip1*^*WT*^ flies (Figure 4A). Furthermore, to evaluate its effect on motor performance, we next measured the activity levels of *dSgip1*^*−/−*^ mutants, wild-type controls, and *dSgip1*^*WT*^ animals using home-built ethoscopes.^37^ In this assay, young flies (5-day-old) were loaded into these devices and movements were automatically recorded and analyzed over a 5-day period. Interestingly, *dSgip1*^*−/−*^ flies performed more micromovements (for example, grooming) than controls. Additionally, these young flies walked significantly shorter distances at a slower pace than controls and *dSgip1*^*WT*^ flies (Figures 4B and 4C). Overall, *dSgip1*^*−/−*^ mutants were less active than wild-type controls or *dSgip1*^*WT*^ animals.

Early-onset parkinsonism caused by mutations in synaptic proteins is often associated with epileptic seizures, which we also observed in subject III:3.^10^^,^^12^ Therefore, we evaluated whether our *dSgip1*^*−/−*^ animals suffer from startle-induced locomotion defects and seizures. When startled (tapped down or briefly vortexed), we noticed that *dSgip1*^*−/−*^ flies increased their speed of movement. However, this startle-induced locomotion resulted in uncoordinated movements and falling, reminiscent of seizure-like behavior. To quantify this, we evoked mechanical stress by vortexing young flies for 10 s and then counted the number of animals showing seizure-like behavior and slow, uncoordinated movements. We found that *dSgip1*^*−/−*^ null mutants were significantly impaired compared to controls and *dSgip1*^*WT*^ animals (Figure 4D).

Seizure-like behavior in flies is often accompanied by increased temperature sensitivity.^38^^,^^39^ To test this, we incubated young flies at high temperature (38°C) and recorded the time it took the flies to become completely paralyzed. Although controls and *dSgip1*^*WT*^ animals did not paralyze in our experimental time frame, *dSgip1*^*−/−*^ null mutant flies were paralyzed within the first ∼55 min of incubation at high temperature (Figure 4E). These results indicate that the loss of dSgip1 function caused locomotor defects in young *Drosophila.*

### Aged *dSgip1*^−/−^ mutants showed widespread neurodegeneration, including dopaminergic synapse loss

**Figure 5:**
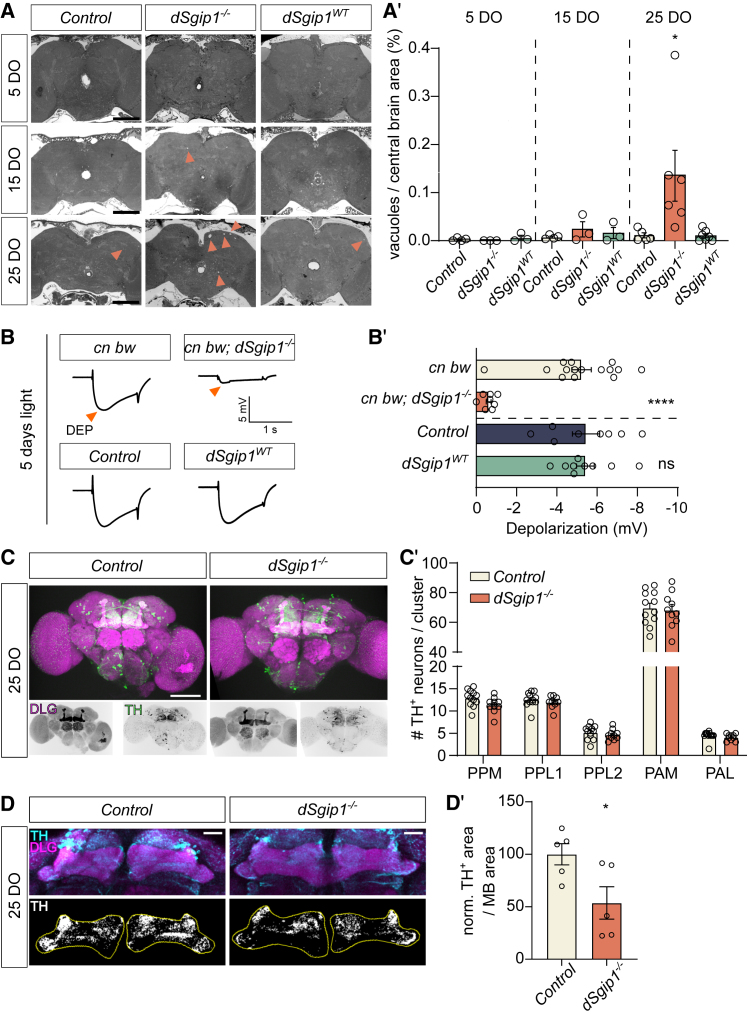
Loss of *dSgip1* induces widespread degeneration, including dopaminergic synapse loss (A) Widefield images of toluidine blue-stained adult brains of the indicated genotypes and ages. Arrowheads indicate degenerative vacuoles. Scale bar: 100 μm. (A′) Quantification of the area occupied by degenerative vacuoles, expressed as percentage of central brain area. Number of analyzed brains ≥ 3 per condition. Statistical significance: two-way ANOVA with Šídák’s multiple comparisons test: ∗*p* < 0.05. Bars: mean ± SEM, and points are individual values. (B and B′) Average ERG traces of flies of the indicated genotypes exposed for 5 days to constant light (B) and quantification of the amplitude of the depolarization as a measure for photoreceptor degeneration (B′). Orange arrowheads indicate depolarization (DEP). Number of recorded animals per genotype ≥ 8. Statistical significance: unpaired t test. Knockout and knockin flies were compared respectively to *cn bw* and control flies. ∗∗∗*p* < 0.001, ∗∗∗∗*p* < 0.0001; ns, not significant. Bars: mean ± SEM, and points are individual values. (C) Representative maximum projection composite confocal images of 25-day-old brains of the indicated genotypes labeled for the post-synaptic marker DLG (magenta) and the dopaminergic marker TH (green). Scale bar: 100 μm. (C′) Quantification of the number of TH^+^ neurons per identified dopaminergic cluster. The number of analyzed brains per genotype ≥ 10. Statistical significance: two-way ANOVA, followed by a *post hoc* Tukey test; ns, not significant. Bars: mean ± SEM, and points are individual values. (D) Top: representative maximum projection composite confocal image that focusses on the dopaminergic innervation (TH, cyan) of the MB of control and *dSgip1*^*−/−*^ fly brains, labeled in (C′). Bottom: thresholded TH^+^ area of middle z-plane section within outlined area of the MB (yellow line, based on the DLG area of the MB, magenta). Scale bar: 20 μm. (D′) Quantification of the dopaminergic synaptic area within the outlined MB area in aged *dSgip1*^*−/−*^ brains relative to the control. The number of analyzed brains per genotype ≥ 5. Statistical significance: unpaired t test: ∗*p* < 0.05. Bars: mean ± SEM, and points are individual values. See also Figure S2.

To assess whether the loss of dSgip1 function is also associated with age-related neurodegeneration, we performed histological sectioning and toluidine staining of heads of young, 5-day-old, and older, 25-day-old, adult flies and quantified the vacuole area (these are regions where the brain degenerated) within the central brain. Although in young flies we did not yet detect a significant amount of degeneration, we observed a gradual increase in the vacuole area in brains of older *dSgip1*^*−/−*^ null mutants (Figures 5A and 5A′) indicating that loss of *dSgip1* caused progressive neurodegeneration.

To further assess the integrity of neuronal function and assess susceptibility to stress, we next recorded electrophysiological responses of the visual system of flies exposed to stress. We engineered control and *dSgip1*^*−/−*^ flies to have white eyes (*cn bw* mutations) such that the application of constant light over several days induced a stressful stimulus. We then placed the animals in constant-light or in constant-dark (control, data not shown) environments. Subsequently, we recorded the response of the visual system to short 1 s light pulses using extracellular voltage recordings (electroretinograms [ERGs]). We found that *dSgip1*^*−/−*^ flies placed in constant light, but not controls nor *dSgip1*^*WT*^ flies, showed a strong reduction in depolarization (DEP) amplitude (Figures 5B and 5B′). This phenotype has previously been amply associated with degeneration of the photoreceptors.^7^^,^^40^ Hence, the loss of *dSgip1* caused light-induced neurodegeneration.

PD associates with dopaminergic neuron dysfunction. Hence, we assessed the integrity of these neurons in (25-day-old) aged control and *dSgip1*^*−/−*^ fly brains using an anti-tyrosine hydroxylase (TH) labeling. While the number of anti-TH-positive (TH^+^) dopaminergic neuronal cell bodies across the different dopaminergic neuron clusters was not affected (Figures 5C and 5C′), the synaptic area of the dopaminergic neurons innervating the mushroom body (the brain structure regulating multiple functions like olfactory learning and memory, sleep, and locomotion^41^^,^^42^^,^^43^^,^^44^^,^^45^) was significantly reduced in *dSgip1*^*−/−*^ mutants compared to controls (Figures 5D and 5D′). Hence, dSgip1 function is required for the maintenance of dopaminergic neuron synaptic integrity in the fly brain.

### Evoked neurotransmission was reduced in *dSgip1* loss-of-function mutants

**Figure 6:**
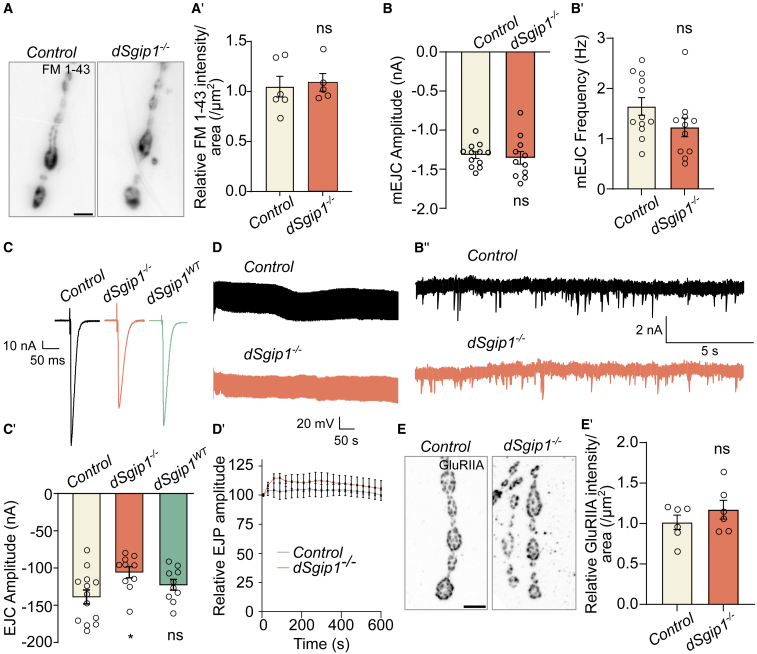
Evoked neurotransmission is impaired in *dSgip1* loss-of-function mutants (A) Widefield images of FM 1-43 fluorescence at NMJs of control and *dSgip1*^*−/−*^ larvae stimulated for 1 min with 90 mM KCl in the presence of 1.5 mM CaCl_2_. Scale bar: 5 μm. (A′) Quantification of FM 1-43 labeling intensity normalized to the NMJ area. 4 NMJs per animal were imaged from ≥5 animals per genotype. Statistical significance: unpaired t test. ns, not significant. Bars: mean ± SEM, and points are individual values. (B and B′) Quantification of the amplitude (B) and frequency (B′) of mEJCs recorded in the presence of 1 mM CaCl_2_ at third-instar larval NMJs of the indicated genotypes to measure spontaneous neurotransmitter release. Number of animals recorded ≥ 11 per genotype. Statistical significance: unpaired t test. ns, not significant. Bars: mean ± SEM, and points are individual values. (B″) Sample mEJC traces (quantified in B and B′). (C and C′) Sample EJC traces (C) and quantification of the EJC amplitude (C′). EJCs were recorded at third-instar larval NMJs at 0.2 Hz stimulation in the presence of 1 mM CaCl_2_. Number of animals recorded ≥ 9 per genotype. Statistical significance: unpaired t test. ∗*p* < 0.05. Bars: mean ± SEM, and points are individual values. (D and D′) Sample EJP traces (D) and quantification of relative EJP amplitudes (D′) of indicated genotypes. EJPs were recorded at third-instar larval NMJs for 600 s during a high-frequency stimulation train (10 Hz) in the presence of 2 mM CaCl_2_. Number of animals recorded ≥ 6 per genotype. Statistical significance: unpaired t test: not significant. Points: mean ± SEM. (E and E′) Maximum projection confocal images of NMJs of third-instar larvae of the indicated genotypes labeled with antibodies against the glutamate receptor GluRIIA (E) and the quantification of the GluRIIA levels normalized to NMJ area (E′). Scale bar: 5 μm. (E′) Statistical significance: unpaired t test. ns, not significant. Bars: mean ± SEM, and points are individual values. See also Figure S3.

Seizure-like behavior in fruit flies can be associated with defects in SV trafficking and neuronal communication.^46^^,^^47^^,^^48^ We tested the ability of *dSgip1*^*−/−*^ mutant synapses to create new SVs by endocytosis. FM 1-43 is a fluorescent lipophilic dye that binds to the neuronal membrane and, upon stimulation, is internalized into newly formed SVs.^49^ Hence, the amount of labeling is a measure of endocytic vesicle formation. We dissected third-instar *Drosophila* larvae to expose the NMJs, stimulated for 1 min with 90 mM KCl in the presence of FM 1-43 and quantified the fluorescence intensity. Although endocytic mutants take up less dye, FM 1-43 labeling was very similar in controls and *dSgip1*^*−/−*^ mutants (Figures 6A and 6A′), indicating that membrane uptake (endocytosis) during this short but strong stimulation paradigm was not affected.

To assess neurotransmission, we measured excitatory junctional currents (EJCs) at NMJs using two-electrode voltage-clamp recordings. Miniature EJCs (mEJCs) are elicited by spontaneous vesicle fusion and neurotransmitter-mediated opening of post-synaptic glutamate receptors. We found similar mEJC amplitudes and frequencies in controls, *dSgip1*^*WT*^, and *dSgip1*^*−/−*^ animals (Figures 6B–6B″). Furthermore, the levels and localization of the post-synaptic glutamate receptor GluRIIA were similar across genotypes (Figures 6E and 6E′). Collectively, this indicated that vesicle recruitment, neurotransmitter vesicle loading and release, and receptor activation were not affected by the loss of dSgip1 function.

We then measured the evoked responses by electrically stimulating the motor neurons under physiological 1 mM calcium concentration at low frequency (0.2 Hz; Figures 6C and 6C′). This experiment showed a significant reduction in the EJC amplitude of *dSgip1*^*−/−*^ animals compared to wild-type and *dSgip1*^*WT*^ controls. Given that the mEJC amplitude was normal, our data indicated that the loss of *dSgip1* caused a significantly lower quantal content (the number of quanta released per stimulation). We further explored this by also recording excitatory junction potentials (EJPs) during prolonged high-frequency stimulation (600 s at 10 Hz) and found that *dSgip1*^*−/−*^ mutants were able to maintain release throughout this period, without increasing or depressing the EJP amplitude (Figures 6D and 6D′). Hence, neurotransmitter release, but not other basic features of synaptic plasticity, was affected by the loss of *dSgip1*.

### The synaptic architecture is largely unchanged in *dSgip1*^−/−^ mutants

To understand why the EJC amplitude was affected, we labeled *dSgip1*^*−/−*^ null mutant NMJs with different synaptic markers, including several SV transmembrane proteins (Synaptobrevin [nSyb], Synaptotagmin 1 [Syt1], vesicular glutamate transporter VGlut1, the vacuolar-type H^+^ ATPase vATPase [Vha-100]), SV-associated proteins (Cysteine String Protein [CSP] and Synapsin), active zone proteins (Syntaxin [Syx1A] and Bruchpilot [Brp]), and endocytic peri-active zone proteins (EndophilinA [EndoA], Synaptojanin [Synj], and Dynamin). We found that the distribution of these proteins was not obviously affected, with Syt1 showing a small and significant reduction in *dSgip1*^*−/−*^ synaptic terminals (Figures S3A and S3B′). Hence, synaptic architecture appeared largely intact in *dSgip1*^*−/−*^ mutants.

Our patients with *SGIP1* mutations manifested with clinical characteristics reminiscent of those affected by *DNAJC6/Auxilin* or *SYNJ1* mutations.^10^^,^^12^^,^^26^ We and others previously showed a genetic interaction between Synj and dAux in flies and mice^13^^,^^50^: overexpression of Synj rescues fly *auxilin* (*dAux*) mutant phenotypes.^13^ Therefore, we tested whether neuronal overexpression (<*nSybGal4*) of Synj or Syt1 could rescue the behavioral defects and neurodegeneration of *dSgip1*^*−/−*^ mutants. However, neither Synj nor Syt1 rescued the paralysis behavior of young (5-day-old) *dSgip1*^*−/−*^ flies, nor did it prevent neurodegeneration in older 25-day-old *dSgip1*^*−/−*^ brains (Figures S2A and S2B′). Therefore, we did not find evidence for a genetic interaction between *dSgip1* and *syt1* or *synj*.

### Lack of multivesicular bodies at *dSgip1*^−/−^ mutant synapses

We next analyzed the ultrastructure of *dSgip1*^*−/−*^ presynaptic terminals by transmission electron microscopy (TEM). In line with our immunohistochemistry analyses (Figures S3A–S3B′), most ultrastructural features appeared normal between the different genotypes, including the presynaptic specializations that dock SVs for release (T-bars) and SV number (<80 nm) (Figures S4A–S4E). We did observe a slightly smaller SV diameter (Figures S4G and S4H) that may be explained by the proposed role of SGIP1 in the construction of the endocytic vesicles.^20^ However, intriguingly, and despite analyzing a substantial number of samples, we were unable to discern multivesicular bodies (MVBs) in *dSgip1*^*−/−*^ mutant synapses, while these organelles were readily detected in controls (Figures S4A–S4C‴ and S4F). MVBs are sorting organelles that are formed by the invagination of endosomal membranes. This creates intralumenal vesicles that contain membrane proteins and cytoplasm. MVBs can fuse with lysosomes and degrade their content or fuse with the plasma membrane and expel their intralumenal vesicles.^51^ Hence, our data suggested a role for dSgip1 in synaptic sorting and proteostasis by regulating MVB function.

**Figure 7:**
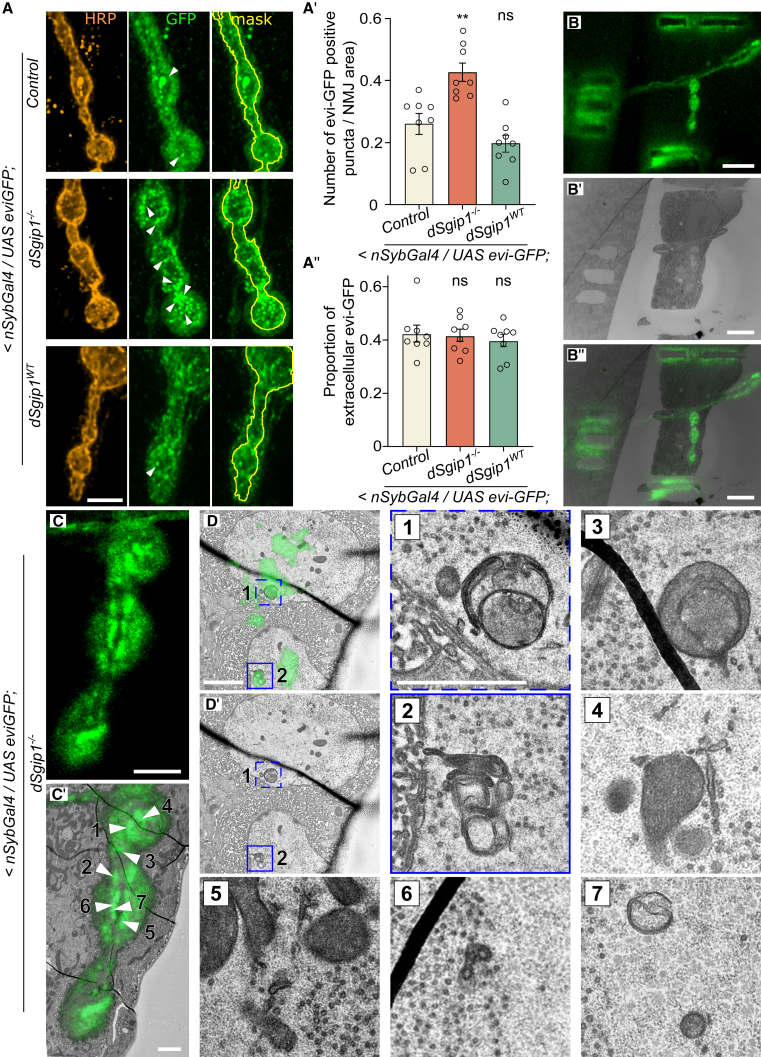
Accumulation of evi-GFP-positive degradative organelles in *dSgip1*^*−/−*^ mutant synapses (A) Maximum intensity projection confocal images of third-instar larval NMJs showing the distribution of neuronally expressed evi-GFP (*<nSybGal4*; green, middle) in the indicated genotypes. Samples were labeled with antibodies against GFP (green) and HRP (orange), a neuronal membrane marker. A mask of HRP is depicted in the evi-GFP channel (yellow line, right). Note that numerous intracellular evi-GFP accumulations (arrowheads) are present in NMJs of *dSgip1*^*−/−*^ animals, while the amount of extracellular evi-GFP puncta (exosomes) is similar at *dSgip1*^*−/−*^, control, and *dSgip1*^*WT*^ NMJs. Scale bar: 5 μm. (A′ and A″) Quantification of the number of intracellular evi-GFP-positive accumulations per NMJ area (A′) and the ratio between internal and released evi-GFP (A″). Bars: mean ± SEM, and points are individual values. 3–4 NMJ per animals were quantified, from ≥8 animals per condition. Statistical significance: one-way ANOVA with Šídák’s multiple comparisons test (A′) and ANOVA Kruskal-Wallis test followed by a Dunn’s *post hoc* test (A″). ns, not significant, ∗∗*p* < 0.01, compared to control. Bars: mean ± SEM, and points are individual values. (B–D′) Correlative light and electron microscopy (CLEM) of *dSgip1*^*−/−*^ mutant NMJs expressing evi-GFP in neurons (*<nSybGal4*). (B–B″) Confocal images of NMJs with evi-GFP fluorescence and near-infrared branding (NIRB)-induced marks around the region of interest (ROI) visible both in TEM and fluorescent mode due to autofluorescence. (C and C′) This marks the string of boutons of interest (C) and allows subsequent detection of the same ROI in the obtained TEM images (C′). (D and D′) Overlay the fluorescent images with the corresponding TEM images. Arrowheads with assigned number indicate the evi-GFP-positive accumulations that were correlated with structures in the obtained serial section TEM images (enlarged images [1–7]). Note that intensities of evi-GFP were adjusted to identify unequivocal structures across multiple TEM sections (see in C′ and D). Scale bars in (B–B″): 20 μm, (C): 5 μm, (C′): 5 μm, (D and D′) and (1–7): 1 μm. Note that evi-GFP-positive accumulations correlated with auto-lysosomal structures and vesicular tubular structures, and not MVBs. See also Figure S4.

To explore this further, we expressed evenness interrupted (evi)-GFP in neurons of *dSgip1*^*−/−*^ mutants and controls (using <*nSybGal4*). Evi is an MVB-membrane-associated protein that also decorates intralumenal MVB vesicles. Therefore, evi-GFP reports on MVB biogenesis and its fate (transport, fusion, …).^52^ Compared to controls, the distribution of this marker was strongly altered at *dSgip1*^*−/−*^ mutant synapses and was visible as abnormal sub-synaptic structures/aggregates (Figures 7A and 7A′). On the contrary, the release of evi-GFP-labeled vesicles into the extracellular space (exosomes) was normal (Figures 7A and 7A″), indicating that this aspect of MVB function was not affected. Given that in our TEM analyses mutant synapses were devoid of MVBs, we wondered what the nature of these evi-GFP-labeled structures was and conducted correlative light and electron microscopy experiments. We localized the evi-GFP fluorescence in our TEM grid based on laser branding marks (Figures 7B–7B″). This enabled us to make one-on-one correlations between the evi-GFP-labeled structure and the synapse ultrastructure (Figures 7B–7D′). The GFP overlap in TEM did not reveal MVBs (consistent with our regular TEM results; Figure S4), but it revealed degradative auto-lysosomal-like structures and vesicular tubular structures (Figure 7, *zooms* 1–7). Given that exosomes were still released in *dSgip1*^*−/−*^ mutants and evi-GFP is readily found in degradative auto-lysosomal organelles, our data are consistent with increased MVB flux in *dSgip1*^*−/−*^ mutants. This suggested that the normal function of dSgip1 is to inhibit MVB-to-plasma membrane and lysosome fusion. Our findings positioned the role of dSgip1 alongside other proteins associated with parkinsonism, specifically in the regulation of synaptic protein and membrane turnover processes.

## Discussion

We present the identification of a loss-of-function variant in *SGIP1* as a plausible candidate gene to cause recessive parkinsonism. Mutations in this gene were not previously identified as a causal gene for this disease. We show that the loss of dSgip1 function causes a defect in synaptic quality control, a phenotype shared by other parkinsonism gene variants.^7^^,^^8^^,^^9^^,^^16^^,^^53^ Indeed, the clinical manifestations of the two affected subjects resembled young-onset parkinsonism, including the additional features of intellectual/cognitive dysfunction and epileptic seizures caused by mutations in *SYNJ1* and *Auxilin/DNAJC6*.^10^^,^^12^ Interestingly, the proteins encoded by these genes are also involved in synaptic quality control.^8^^,^^13^^,^^50^^,^^54^ The FDG PET scans further identified the typical metabolic features of PD at the regional and network levels. Both patients had elevated PDRP expression (scores > +1.5), PDRP predominance (delta > +1.0), and image-based classification as idiopathic PD with high probability (>99%). Thus, diagnostic alternatives such as an atypical parkinsonian look-alike syndrome were unlikely in both patients.

While we await further replication in independent kindred, the likely pathogenicity of the *SGIP1* variant is supported by several lines of evidence. In direct sequencing, we did not detect pathogenic mutations in the other familial recessive parkinsonian genes *PARK2*, *PLA2G6*, and *DNAJC6*. Further whole-exome sequencing also excluded pathogenic variants in *PINK1*, *DJ1*, *ATP13A2*, *FBXO7*, *VPS13C*, *SYNJ1*, *PODXL*, and other known genes associated with early-onset parkinsonism. Homozygosity mapping ultimately only supported *SGIP1* as the candidate gene within the shared genomic coordinates of the same cytogenetic location; the mutation we found in *SGIP1* (c.2080T>G (p.W694G)) is absent from the other variation databases. The mutated tryptophan at position 694 is also well conserved across species, suggesting that it is critical for protein function. *In silico* and *in vivo* analyses agree that the W694G mutation destabilizes the core of the μHD. Further *in vivo* studies indeed show that loss of *dSgip1* function causes numerous neurological problems at the organismal level, including severe behavioral and motor dysfunction and seizure-like behavior and progressive neurodegeneration, including dopaminergic synapse loss. These defects coincided with synaptic transmission problems and protein quality control defects likely elicited by progressive MVB flux, causing MVB degradation or expedited fusion with the plasma membrane. This provides further experimental evidence that the *SGIP1* variant is disease-causing through a loss-of-function mechanism.

Our observation adds to the growing body of evidence for the crucial role of synaptic proteins including SH3GL2/EndoA1, Auxilin, and Synj1, in the development of recessive parkinsonism.^10^^,^^11^^,^^12^^,^^17^^,^^26^ Previous work by us and others has shown physical and functional interactions between several of the proteins encoded by these genes (i.e., SGIP1 binds EndoA1 and Synj1 binds EndoA1)^19^^,^^55^ and genetic interactions (i.e., Synj rescues Aux and EndoA1).^13^^,^^56^ While we did not find a genetic interaction between *synj* and *dSgip1* (possibly because we used a *dSgip1*^*−/−*^ null mutant), all these proteins have partially overlapping synaptic functions. Not only do they play a role in SV recycling and/or uncoating following endocytosis^20^^,^^34^^,^^56^^,^^57^^,^^58^^,^^59^^,^^60^ but also pathogenic mutations in these proteins primarily affect synaptic proteostasis, including synaptic autophagy and endo-lysosomal function.^7^^,^^8^^,^^16^^,^^54^^,^^61^ Now, we show that *dSgip1*^*−/−*^ mutants lack synaptic MVBs that are crucial organelles in the proteostasis and protein/membrane quality control network. These organelles are formed by the invagination of endosomes, and their contents can be degraded by lysosomes or expunged from cells by plasma-membrane fusion.^51^ The lack of MVBs in *dSgip1*^*−/−*^ mutants is consistent with increased MVB-to-membrane and lysosome fusion, and this may disrupt normal maturation and protein degradation. In this context, it is interesting that SGIP1 can interact with PI(3)P, a phosphoinositide lipid found on endosomes,^20^ and with other phosphoinositides in SVs. Hence, SGIP1, like other early-onset Parkinson’s proteins, is associated with functions that regulate synaptic proteostasis. Additional studies will now be required to unravel the potential convergent mechanistic pathways between SGIP1 and these other synaptic proteins related to Parkinson’s disease.

SGIP1 was originally identified as an EndoA1 interactor.^19^ Subsequently, it was reported to be involved in the early steps of membrane retrieval during endocytosis via its membrane phospholipid binding domain and through interaction with Intersectin 1 and Eps15.^20^^,^^21^ However, membrane retrieval measured by dye uptake experiments and neurotransmitter release during intense stimulation, a process that requires intense vesicle retrieval, did not show obvious defects. Similarly, and unlike observations in mutants that affect SV endocytosis,^56^^,^^58^^,^^62^^,^^63^^,^^64^ SV abundance monitored by electron microscopy was normal. We did measure slightly smaller vesicle diameters that are possibly consistent with (minor) disruptions in the vesicle retrieval machinery,^65^ while the quantal size (mEJC amplitude) and the abundance of vGlut1, the vATPase that acidifies the lumen of the vesicle and the post-synaptic glutamate receptor, were not affected. Taken together, this suggests that SV formation and transmitter loading were largely unaffected.^66^^,^^67^^,^^68^^,^^69^^,^^70^ Instead, we found a profound depletion of synaptic MVBs, suggesting membrane trafficking and protein/lipid sorting problems downstream of vesicle formation at the plasma membrane. It is possible that these quality control problems eventually culminate in a lower EJC amplitude, also explaining the behavioral deficits such as seizures and motor dysfunction^46^^,^^47^ and ultimately neurodegeneration.

In conclusion, the results of our genetic analysis and functional studies delineate the loss-of-function *SGIP1* mutation as the most probable cause of a form of recessive parkinsonism with significant implications for the diagnosis of the disease, genetic counseling, and pharmacological treatment. Our functional/experimental data provide additional evidence for the crucial role of synaptic dysfunction and proteostasis impairment in the pathophysiology of recessive parkinsonism.

### Limitations of the study

We limited our examination of phenotypic characteristics to a single Arab family identifying a rare mutation. This family is isolated and inbred, possibly explaining why we failed, thus far, to identify additional families or individuals with *SGIP1* mutations. It will, nonetheless, be essential to verify the presence of *SGIP1* variants in additional cases of familial parkinsonism to provide definite proof of causality to disease. Although other genes linked to early-onset parkinsonism are also rare, collectively, their functions seem to converge on similar synaptic proteostasis pathways, as does the function of SGIP1. Considering the rarity of this gene variant and our extensive clinical experience, during which we have not encountered or observed similar cases in three different countries—Canada, India, and Middle East Asia—we expect that further genotypic characterization of the same or new *SGIP1* pathogenic variants may be a long process.

*In silico* analyses, of the *SGIP1* variant we found, suggest it is pathogenic and causes a loss of protein function (based on the folding problems of the mutant protein). We therefore modeled this by creating knockout fruit flies, identifying synaptic function defects, behavioral problems, and defective dopaminergic neuron innervation, indicating *SGIP1* is required for normal dopaminergic neuron maintenance. While this approach is valid to understand the effects of loss of SGIP1 function, it lacks possible effects that are specific to the mutant protein, and it also does not take a human-specific context into account. Future work could involve expressing the human mutant protein in flies or creating human-induced neurons *in vitro*, enabling further analyses of this protein variant.

## Resource availability

### Lead contact

Further information and requests for resources and reagents should be directed to the lead contact Patrik Verstreken (patrik.verstreken@kuleuven.be).

### Materials availability

All reagents/materials generated in this study will be made available upon request. The request may require a completed Material Transfer Agreement.

### Data and code availability

• Individual-level sequencing data from this study are not publicly available due to participant’s consent restrictions and privacy concerns; however, requests from accredited researchers for access to the next-generation sequencing (NGS) data relevant to this manuscript can be made by contacting Patrick Scott (patrick.scott.hsj@ssss.gouv.qc.ca). The clinical data reported in this study will be shared by Ramachandiran Nandhagopal (rnandagopal@yahoo.com) upon request. All other data reported in this paper will be shared by the lead contact upon request.
• Any additional information required to reanalyze the data reported in this paper is available from the lead contact upon request.
• This paper does not report original code.

## Acknowledgments

We thank subjects III:1 and III:3 and their family for their participation in this research study. Furthermore, we thank the Bloomington *Drosophila* Stock Center (NIH P40OD018537) for providing flies and BestGene Inc for *Drosophila* embryo injections and the generation of transgenic strains. We also thank the technology units at the VIB-KU Leuven Center for Brain & Disease Research, the VIB core facilities, and the cell and tissue imaging cluster at KU Leuven (supported by Hercules AKUL/11/37 and FWO G.0929.15 to Pieter Vanden Berghe). The authors also thank Irka Van de Gaer for fly husbandry and lab support and other members of the Verstreken lab for helpful discussions and advice. This work was supported by 10.13039/501100004727VIB, FWO Vlaanderen, ERC, the 10.13039/100014989Chan Zuckerberg Initiative, a Methusalem grant of the 10.13039/501100011878Flemish Government, Opening the Future (Leuven University fund to P.V.), and the Research Council of Oman (RC/MED/GENT/14/01 to P.S.). E.N. was supported by the 10.13039/501100003130FWO postdoc fellowship 1282123N. N.K. and R.P. were supported by an 10.13039/501100003043EMBO long-term postdoctoral fellowship. P.V. is an alumnus of the FENS-Kavli Network of Excellence.

## Author contributions

Conceptualization: M.D., S.K., P.V., P.S., and R.N. Methodology: M.D., S.K., P.V., P.S., and R.N. Investigations: M.D., S.K., C.C., J.S., S.F.G., N.K., E.N., R.P., N.S., R.N., A.A.A., P.S., S.A.A., D.M., C.C.T., and D.E. Writing: M.D., S.K., R.N., and P.V. Funding acquisition: P.S., C.C., N.K., and P.V. Supervision: P.V. and R.N.

R.N. and A.A.A. performed and are responsible for the clinical and neurological examination of the patients; P.S. performed and is responsible for the genetic examination and analyses; S.A.A. performed the neuropsychological assessment of the cases; D.M. obtained the pedigree tree and consent of the family for participation in the study and consent for publication; C.C.T. and D.E. performed the metabolic network analysis of FDG PET scans; E.N. and R.P. assisted in the structural protein analysis; S.K., C.C., J.S., S.F.G., N.K., N.S., M.D., and P.V. performed and are responsible for the *Drosophila* experiments, including behavior, electrophysiology, molecular genetics, histology, and electron microscopy.

## Declaration of interests

The authors declare no competing interests.

## STAR★Methods

### Key resources table

**Table:** 

REAGENT or RESOURCE	SOURCE	IDENTIFIER
**Antibodies**		

guinea pig anti-EndoA (GP69)	Verstreken et al.^58^	N/A
rabbit anti-GFP	Invitrogen	Cat#: A11122; RRID: AB_221569
goat anti-GFP	Abcam	Cat#: ab5450; RRID: AB_304897
mouse anti-Brp	DSHB	Cat#: nc82; RRID: AB_2314866
mouse anti-Syntaxin1A	DSHB	Cat#: 8C3; RRID: AB_528484
mouse anti-CSP	DSHB	Cat#: ab49; RRID: AB_2307345
mouse anti-GluRIIA	DSHB	Cat#: 8B4D2; RRID: AB_528269
mouse anti-Synaptotagmin1	DSHB	Cat#:3H2 2D7; RRID: AB_528483
rabbit anti-Synaptojanin	Verstreken et al.^56^	N/A
guinea pig anti-Vha100-1	Gift from Robin Hiesinger^71^	N/A
mouse anti-Synapsin	DSHB	Cat#: 3C11; RRID: AB_528479
mouse anti-Dynamin	DB Biosciences	Cat#: 610246; RRID: AB_397641
rat anti-Synaptobrevin	Gift from Hugo Bellen	N/A
mouse anti-DLG	DSHB	Cat#: 4F3; RRID: AB_528203
rabbit anti-Tyrosine Hydroxylase (TH)	Millipore	Cat#: AB152; RRID: AB_390204
rabbit anti-GAPDH	Thermo Fisher	Cat#: PA1-16777; RRID: AB_568552
rabbit anti-HRP	Jackson Immuno Research	Cat#: 323-005-021; RRID: AB_2314648
Alexa Fluor 488 goat anti-rabbit	Invitrogen	Cat#: A11034; RRID: AB_2576217
Alexa Fluor 555 goat anti-rabbit	Invitrogen	Cat#: A27039; RRID: AB_2536100
Alexa Fluor 647 goat anti-mouse	Invitrogen	Cat#: A21236; RRID: AB_2535805
Alexa Fluor 555 goat anti-mouse	Invitrogen	Cat#: A21422; RRID: AB_141780
Alexa Fluor 647 goat anti-guinea pig	Invitrogen	Cat#: A21450; RRID: AB_2735091
Alexa Fluor 488 goat anti-rat	Invitrogen	Cat#: A11006; RRID: AB_2534074
Alexa Fluor 647 anti-goat	Invitrogen	Cat#: A21447; RRID: AB_2535864

**Chemicals, peptides, and recombinant proteins**		

FM1–43 Dye (N-(3-Triethylammoniumpropyl)-4-(4-(Dibutylamino) Styryl) Pyridinium Dibromide)	Invitrogen	Cat#: T3163

**Critical commercial assays**		

Maxwell RSC microRNA tissue kit	Promega	Cat#: AS1460
SuperScript III First-Strand Synthesis System for RT-PCR	Thermo Fisher	Cat#: 18080051
Quick Start Bradford Protein Assay kit	Bio-Rad	Cat#: 5000202
Ion Ampliseq Exome Kit	Thermo Fisher	Cat#: A38264
CytoScan™ HD Array Kit	Thermo Fisher	Cat#: 901835
Ampli Taq Gold Fast PCR Master	Thermo Fisher	Cat#: 4390941
Big Dye v3.1 Cycle Sequencing kit	Thermo Fisher	Cat#: 4337455

**Experimental models: organisms/strains**		

*D. melanogaster*: w^1118^	BDSC	RRID: BDSC_3605; Fly base: FBal0018186
*D. melanogaster*: GMR57C10-Gal4	BDSC	RRID: BDSC_39171; Fly base: FBti0137043
*D. melanogaster: cn bw*	BDSC	RRID: BDSC_264; Fly base: FBst0000264
*D. melanogaster*: UAS-Evi-GFP	Gift from Michael Boutrons^72^	N/A
*D. melanogaster*: UAS-DF-Syt1	Gift from Robin Hiesinger^73^	N/A
*D. melanogaster*: *y[1] w[67c23]; Mi{PT-GFSTF.2}VGlut[MI04979-GFSTF.2]*	BDSC^74^	RRID: BDSC_59411
*D. melanogaster:* CG8176^-^	This study	N/A
*D. melanogaster:* CG8176 WT	This study	N/A
*D. melanogaster:* UAS-GFP-CG8176 WT	This study	N/A
*D. melanogaster:* UAS-GFP-CG8176 WG	This study	N/A
Fly lines used in this study, see Table S4	–	N/A

**Oligonucleotides**		

Primers for RT-PCR, see Table S4	This study	N/A
Primers for cloning, see Table S4	This study	N/A
Homology arms, see Table S4	This study	N/A
gBlocks for cloning, see Table S4	This study	N/A

**Recombinant DNA**		

pCFD4	Gift from Simon Bullock^75^	RRID: Addgene_49411
pCFD4_gRNA	This study	N/A
pWhite-STAR	Choi et al.^76^	N/A
pWhiteSTAR_dSgip1	This Study	N/A
pUC19	Addgene	RRID:Addgene_50005
pReC_dSgip1-WT	This Study	N/A
pUAST attB w+	Bischof at al.^77^	N/A
pUAST-GFP-dSgip1-WT	This Study	N/A
pUAST-GFP-dSgip1-WG	This Study	N/A

**Software and algorithms**		

GraphPad Prism 9	GraphPad Software	https://www.graphpad.com/scientific-software/prism/
ImageJ-win64	National Institute of Health	https://imagej.nih.gov/ij/
Inkscape 1.1.2	Inkscape’s Contributors	https://inkscape.org/de/release/inkscape-1.1.2/
NIS-Elements	Nikon	https://www.microscope.healthcare.nikon.com/
Zen black	Zeiss	https://www.zeiss.com/
Axoscope 10.7	Molecular Devices	https://support.moleculardevices.com/s/article/Molecular-Devices-Software
Igor Pro 6.37	Wave Metrics	https://www.wavemetrics.com/
Axoclamp900A	Molecular Devices	https://support.moleculardevices.com/s/article/Molecular-Devices-Software
Clampfit 10.7	Molecular Devices	https://support.moleculardevices.com/s/article/Molecular-Devices-Software
R Studio 3.6.3	The R Project	https://www.r-project.org/
GIMP 2.10.30	GIMP	https://www.gimp.org/
CLC workbench 22	Qiagen	https://digitalinsights.qiagen.com/products/clc-main-workbench-direct-download/
LAS v4.0	Leica	https://www.leica-microsystems.com/
LightCycler 480 Software	Roche	https://diagnostics.roche.com/
Statistical Parametric Mapping	Functional Imaging Laboratory	https://www.fil.ion.ucl.ac.uk/spm/
MATLAB 7.3	MathWorks	https://www.mathworks.com/?s_tid=gn_logo
ScanVP	Feinstein Institutes for Neuroscience	https://feinsteinneuroscience.org/
Ion Torrent Suit	Thermo Fisher	https://www.thermofisher.com/it/en/home/life-science/sequencing/next-generation-sequencing/ion-torrent-next-generation-sequencing-workflow/ion-torrent-next-generation-sequencing-data-analysis-workflow/ion-torrent-suite-software.html

### Experimental model and study participant details

#### Subjects

We identified two affected sisters (subjects III:1 and III:3 born of consanguineous Arab parentage) manifesting with young-onset parkinsonism. These subjects underwent comprehensive neurological assessment, neuropsychological testing, biochemical studies, brain magnetic resonance imaging and [^18^F]-Fluorodeoxyglucose Positron Emission Tomography (FDG PET). Following informed consent, blood samples were collected from the affected subjects and their healthy mother and salivary samples from their healthy father and other siblings (subjects III:1, III:3, II:4, II:5, III:7 and III:8). This study was approved by the Institutional Ethics Committee (SQU-EC/158/14).

#### Fly stocks and maintenance

To further investigate the pathogenic nature of the variant, we performed functional studies in fruit flies. Fruit flies were grown on standard cornmeal and sugar beet syrup medium at 25°C. The *dSgip1*^*−/−*^ null mutant, *dSgip1*^*WT*^, *UAS-GFP-dSgip1*^*WT*^ and *UAS-GFP-dSgip1*^*WG*^ flies were generated using strategies described in the method details section related to the *Drosophila* experiments. UAS-DF-Syt1 flies were a gift from Robin Hiesinger.^73^ The *UAS-evi-GFP* flies were a kind gift from Pr. Michael Boutros (DKFZ, Germany).^72^ *y[1] w[67c23]; Mi{PT-GFSTF.2}VGlut[MI04979-GFSTF.2]* were purchased from Bloomington Drosophila Stock Center.^74^ Both male and female flies were included in the study. Based on the experiment, either larvae or adult insects were used, as specified in the appropriate section. The genotypes used in this study are listed in Table S4.

### Method details

#### FDG PET, image processing, and single-case analysis with Statistical Parametric Mapping

Both patients were scanned with FDG PET under resting conditions. All anti-parkinsonian medications were withheld at least 12 h before imaging. PET imaging was performed using a Siemens PET CT scanner following standard protocol. The PET images of the affected patients were spatially normalized and smoothed (FWHM 10 × 10 × 10 mm) using Statistical Parametric Mapping (SPM5, Wellcome Trust Center for Neuroimaging, London, UK) running in MATLAB 7.3 (Mathworks, Sherborn, MA). Using single-case voxel wise analysis with SPM,^78^ we compared each patient’s FDG PET scan to those of an age-matched healthy control (HC) group of 18 subjects, acquired at the Feinstein Institutes for Medical Research, (10 male/8 female; Age: 26.6 ± 4.0; range 20.3–32.8), to show abnormally increased or decreased glucose metabolism in the brain of each patient relative to the HC group.

#### Network analysis

Network analysis, or spatial covariance analysis, of metabolic images can provide an unbiased measurement of functional changes in the whole brain. Using this analysis, we have previously identified and validated spatial covariance metabolic patterns specifically related to PD motor and cognitive abnormalities (termed PDRP and PDCP, respectively).^79^ Moreover, the difference between PDRP and PDCP expression values, termed Delta, was found to be positive in the majority of patients with idiopathic PD.^23^ We also identified specific disease-related metabolic patterns for atypical parkinsonian syndromes (APS), such as multiple system atrophy (MSA) and progressive supranuclear palsy (PSP) (termed MSARP and PSPRP respectively).^80^ In this study, we computed expression values (subject scores) of each of these metabolic patterns in the scans of the two patients and the 18 HC subjects using ScanVP software (freely available upon request at https://feinsteinneuroscience.org). We additionally calculated the delta values (i.e., PDRP score – PDCP score) for both patients.

#### Automated differential diagnosis analysis

We previously developed an automated differential diagnosis algorithm based on disease-related metabolic patterns (PDRP, MSARP, and PSPRP) and the FDG PET scan data of a cohort of American patients.^81^ The algorithm was used to differentiate patients with idiopathic PD from those with atypical parkinsonian syndromes (APS), such as MSA and PSP. This algorithm has been validated in several independent patient cohorts from India,^82^ Slovenia,^83^ and Sweden.^24^ In this study, we applied the algorithm to the subject scores of PDRP, MSARP, and PSPRP to classify each patient as PD or APS (MSA or PSP).

#### Targeted molecular genetic analysis

Genomic DNA was isolated from whole blood samples following standard procedures. Prior to whole exome sequencing, direct Sanger sequencing of *PARK2*, *PLA2G6*, and *DNAJC6* was performed separately in the commercial diagnostic laboratories for the detection of pathogenic variants, including dosage analysis for large deletions and/or duplications.

#### Whole-exome sequencing (WES)

WES was performed on the Ion Torrent Proton sequencer using the capture Ion Ampliseq Exome Kit and Ion Hi-Q sequencing chemistry run on the PI chip v3. Data analysis was performed with the ion Torrent suite of software, including the Ion reporter for variant annotation and filtering (Thermo Fisher Scientific) against the human reference genome assembly 19 (GRCh37). The filtering of variations was based on the allele frequency (MAF ≤0.01), variant predicted effect (excluding synonymous variants) and gene location (coding and exon-intron boundaries). Variants present in the shared region of homozygosity (ROH) between the two affected subjects as determined by genotyping on the CytoScan HD array platform as per the manufacturer’s protocol (Affymetrix, Santa Clara, USA) were prioritised, given the presence of consanguinity.

#### SGIP1 sanger sequencing

Amplicons of the target region within exon 22 of the *SGIP1* gene (GeneBank: NM_032291.4) harboring the c.2080T>G variant were generated using the Ampli Taq Gold Fast PCR Master (Thermo Fisher Scientific). Bi-directional Sanger sequencing for confirmation and segregation within the family was performed using the Big Dye v3.1 sequencing chemistry (Thermo Fisher Scientific). Fragments were separated on the 3500 Genetic Analyser (Thermo Fisher Scientific).

#### Protein structure analysis

The AlphaFold^31^ structure of human wild-type SGIP1 was downloaded from Uniprot (Uniprot: AF-Q9BQI5-F1-v4). The AlphaFold structure of mutant SGIP1^WG^ was predicted by entering its protein sequence in ColabFold,^84^ a platform that offers accelerated prediction of protein structures and complexes by combining the fast homology search of MMseqs2 with AlphaFold2 (Developed by Google DeepMind and EMBL-EBI). Next, both AlphaFold structures were visualized in ChimeraX (v1.6.1; UCSF). The μHD from position 531–828 of wild-type and mutant SGIP1 were analyzed and hydrophobic contacts were determined. AlphaMissense^33^ (AM) (Google DeepMind) was used to predict pathogenicity for all single–amino acid substitutions along the SGIP1 protein sequence (Uniprot: Q9BQI5-1). The average AM pathogenicity for each residue was calculated and plotted as bars. High scores (≥0.564) are represented as likely pathogenic (red), low scores (<0.340) as likely benign (blue) and scores between 0.340 and 0.564 are marked ambiguous or uncertain (gray).

#### Plasmid generation

Primers, gRNA, homology arms and gBlocks are listed in Table S4.

##### pCFD4_gRNA

pCFD4: U6:1-gRNA U6:3-gRNA (gift from Simon Bullock (Addgene plasmid #49411; http://n2t.net/addgene:49411; RRID: Addgene_49411))^75^ was linearized with BbSI and unique gRNA for dSgip1 were cloned into the linearized vector by Gibson Assembly using the primers CRISPR_dSgip1_Fw and CRISPR_dSgip1_Rc. The cloning strategy was based on an established protocol: http://www.crisprflydesign.org/wp-content/uploads/2014/06/Cloning-with-pCFD4.pdf.

gRNA was identified by http://crispr.mit.edu/.

##### pWhiteSTAR_dSgip1

Homology arms of 1 kB surrounding the first common exon (exon 1) of the different transcripts of the gene of interest were cloned into pWhite-STAR.^36^^,^^76^ pWhite-STAR was first linearized with AvrII to insert the right homology arm (RHA) and subsequently digested with XhoI to insert the left homology arm (LHA). The homology arms were amplified by PCR from genomic fly DNA of the target genotype (control fly line: *CS*^*w1118*^). The homology arms were inserted by Gibson Assembly with the following primers: RHA_Fw, RHA_Rc, LHA_Fw and LHA_Rc.

##### pReC_dSgip1-WT

pReC was generated by linearizing pUC19 with SapI and EcoRI followed by insertion of two gBlocks: attB-MCS-L and MCS-attB-R.^36^ pReC was linearized with XhoI and XBaI to insert the 5′UTR of dSgip1 by Gibson Assembly using primers Fw_dSgip1_UTR and Rc_dSgip1_UTR. The resulting plasmid was subsequently digested with SapI to insert the cDNA of dSgip1 by Gibson Assembly using two gBlocks: cDNA_dSgip1_Part 1 and cDNA_dSgip1_Part 2.

##### pUAST-GFP-dSgip1-WT and pUAST-GFP-dSgip1-WG

pUAST attB w+^77^ was linearized with EcoRI and XhoI. eGFP, followed by a short flexible linker, was inserted at the N-terminal of dsgip1-WT or dSGIP1-WG cDNA. The following primers were used for the assembly: Fw_eGFP-dSgip1_Hifi, Fw2_eGFP-dSgip1_Hifi, Rc_eGFP-dSgip1_Hifi and Rc2_eGFP-dSgip1_Hifi.

#### Fly line generation

*dSgip1*^*−/−*^ null mutant flies were generated at Bestgene Inc using the CRISPR/Cas9 system according to the targeting strategy recently described.^36^ Both the tandem gRNA-expressing plasmid pCFD4_gRNA and the donor plasmid pWhiteSTAR_dSgip1 were injected in embryos of flies expressing vas-Cas9(III) (BDSC #51324). This donor plasmid contained an Integrase mediated exchange (IMCE) cassette that expresses mini-white upon genomic integration. Additionally, the IMCE cassette was surrounded by two homology arms to facilitate homology directed repair (HDR). Two double-strand breaks were introduced in the DNA surrounding the first exon, which is shared by all possible transcripts of *dSgip1*. Accordingly, through HDR this exon was replaced with the IMCE cassette. The homology arms were chosen such that the IMCE cassette resided between two non-evolutionarily conserved regions.

*dSgip1*^*WT*^ flies were generated in-house by injecting the rescue plasmid pReC_dSgip1-WT and a plasmid expressing the PhiC31 integrase in embryos of *dSgip1*^*−/−*^ null mutant flies according to the knock-in strategy recently described.^36^ Through PhiC31 integrase mediated cassette exchange the mini-white IMCE-cassette was replaced with the CDS of dSgip1-WT.

*UAS-GFP-dSgip1*^*WT*^ and *UAS-GFP-dSgip1*^*WG*^ flies were generated by in-house injection of respectively the pUAST-GFP-dSgip1-WT or the pUAST-GFP-dSgip1-WG plasmid and a plasmid expressing the PhiC31 integrase. By PhiC31 integrase mediated cassette exchange the constructs were inserted at the locus su(Hw)attP5.

#### Immunohistochemistry and confocal imaging

Third-instar larvae were dissected in cold Ca^2+^ free HL3 (110 mM NaCl, 5 mM KCl, 10 mM NaHCO_3_, 5 mM HEPES, 30 mM sucrose, 5 mM trehalose, and 10 mM MgCl_2_, pH 7.2;^85^) and fixed for 20 min at room temperature with 4% paraformaldehyde or for 5 min with 100% Bouins. Fixed larvae were permeabilized with 0.4% PBX (Triton X-100 in 1X PBS), blocked for 1 h with 10% normal goat serum in PBX and incubated overnight at 4°C with primary antibodies. After several washes, larval filets were incubated with secondary antibodies for 90 min at room temperature. Samples were mounted in Vectashield (Vector Laboratories).

Fly brains of 25-day-old flies were dissected in cold PBS and fixed in 4% paraformaldehyde for 20 min at RT and blocked for 1 h with 5% normal goat serum (MP Biomedicals) in 0.4% Triton X-100 in PBS. Primary antibodies were incubated at 4°C for 1.5 days and secondary antibodies at 4°C for 1 day.

The following antibodies were used: guinea pig anti-EndoA (GP69) [1:2000],^58^ rabbit anti-GFP [1:1000 (Invitrogen)], mouse anti-Brp [1:50 (DSHB)], mouse anti-Syntaxin1A [1:50 (DSHB)], mouse anti-CSP [1:50 (DSHB)], mouse anti-GluRIIA [1:100 (DSHB)], mouse anti-Synaptotagmin1 [1:50 (DSHB)], rabbit anti-Synaptojanin [1:500],^56^ guinea pig anti-Vha100-1 [1:2000 (gift from Robin Hiesinger^71^], mouse anti-Synapsin [1:100 (DSHB)], mouse anti-Dynamin [1:500 (BD Biosciences)], rat anti-Synaptobrevin [1:1000 (gift from Hugo Bellen)], mouse anti-DLG [1:50 (DSHB)], rabbit anti-HRP [1:1000 (Jackson ImmunoResearch)], rabbit anti-TH [1:200 (Millipore)]. Alexa Fluor 488/Alexa Fluor 555 conjugated secondary antibodies [1:1000 (Invitrogen)].

Larval samples (except evi-GFP experiments) were imaged on a Nikon A1R confocal microscope with a 60X (NA 1.4) oil lens and acquired using a Galvano scanner, a zoom factor of 3, line averaging of 2 and step intervals of 0.45 μm. Evi-GFP experiments were also imaged on a Nikon A1R confocal microscope with a Plan Apo VC 60X WI DIC N2 lens and acquired in resonant mode with a zoom factor of 4, line averaging of 16 and step intervals of 0.5 μm. All images were acquired with a pinhole of 1 Airy unit and a resolution of 1024 × 1024 using the NIS Elements software (Nikon). Z-stacks were used in data acquisition and the same image settings were maintained across the genotypes. Confocal images (fluorescence intensities, evi-GFP accumulations and NMJ area) were quantified with ImageJ. Evi-GFP release was quantified using Fiji and R studio.^86^ First, the GFP signal was separated into intracellular and extracellular signals by using the neuronal membrane marker HRP signal to define an intracellular mask. Next, extracellular evi-GFP levels were measured by selecting an area corresponding to a 1 μm dilation around the HRP mask.

Larvae stained for sub-synaptic localization of dSgip1 (GFP-dSgip1) and Brp were imaged on a Zeiss LSM 880 (Airy Scan detector enabled) with a 63X lens (NA 1.4). Zen Black software (2012, Carl Zeiss) was used for image acquisition.

Imaging of adult brains was performed on a Nikon A1R confocal microscope with a 20X (NA 0.95) water immersion lens using a Galvano scanner with line averaging of 2. Z-stacks were acquired with a pinhole of 1 Airy unit, a resolution of 1024 × 1024 and a step interval of 2 μm. First, the total number of TH-positive (TH^+^) dopaminergic neurons was counted manually in both hemispheres for the PPM1, PPM2, PPM3, and PPL1 clusters throughout the brain. Next, the synaptic area of the dopaminergic neurons that innervate the mushroom body (MB) was quantified as follows: in the anti-DLG channel, the outline of the MB was determined in the sum projection of the five z-planes where the synaptic region is located. Subsequently, the anti-TH fluorescence in this area was thresholded (default threshold in Fiji), excluding the background signal, similar to the control. For every brain individually, the area of the TH^+^ thresholded signal was quantified in every plane, summed and normalized to the area of the outlined MB region (TH^+^ area/MB area). Furthermore, for every experiment, the values of the individual TH^+^ area/MB area were normalized to the mean of the control. For representative images, the maximum projection of five z-planes and the thresholded middle z-plane is shown.

#### Western blot

Flies collected separately from three independent crosses were decapitated and heads homogenized with a motorized pestle in lysis buffer (25 mM HEPES, 100 mM NaCl, 1 mM CaCl_2_, 1% Triton X-100, 1X Complete Protease Inhibitor (Sigma)). After incubation on ice for 30 min, samples were centrifuged at 10000 g for 10 min and supernatant collected and quantified by Bradford assay (BioRad) in a GloMax Multi Detection Plate Reader (Promega). After boiling in 1X Laemmli buffer with 8% 2-mercapto-ethanol (Sigma), samples were run on a NuPage 4–12% Bis-Tris gel (Thermo Fisher Scientific), transferred on a nitrocellulose membrane (BioRad) and subsequently blocked with 10% BSA in Tris-buffered saline (TBS). Primary antibodies were incubated overnight at 4°C in antibody solution (5% BSA in TBS supplemented with 0.05% Tween 20). Fluorescent secondary antibodies were incubated for 1 h at room temperature in antibody solution. After detection with the iBright imaging system (Thermo Fisher Scientific), fluorescent bands were quantified in ImageJ. GFP fluorescence was normalized to GAPDH fluorescence.

The following antibodies were used: rabbit anti-GAPDH [1:2000 (Invitrogen)], goat anti-GFP [1:1000 (Abcam)] and Alexa Fluor 488/Alexa Fluor 647 conjugated secondary antibodies [1:1000 (Invitrogen)].

#### RNA extraction, retro transcription, and quantitative real-time PCR (qRT-PCR)

RNA was extracted from # fly heads per genotype in three biological replicates using the Maxwell RSC microRNA tissue kit (Promega) according to the manufacturer’s instructions. 1 μg of RNA was retrotranscribed for each sample (mock samples were included in the retro transcription). First, the RNA was incubated with OligoDT at 65°C for 5 min. After adding the reaction mix (RT buffer, MgCl_2_, DTT, RNase out, dNTP’s and SuperScript III reverse transcriptase (Thermo Fisher Scientific)), samples were incubated 50 min at 50°C and then 5 min at 85°C. Samples were chilled on ice and RNaseH was added before a final incubation at 37°C for 20 min.

Before use, the efficiency of the primer sets to measure mRNA levels of *dSGIP1* (CG8176) was tested by running a qRT-PCR with Light Cycler 480 SYBR Green (Roche) on a serial dilution of the cDNAs. ΔCt were calculated based on Ct values of the housekeeping gene Rp49. Primers are listed in Table S4.

#### Survival analysis

Flies of both sexes were collected as virgin and kept in single tubes. Flies were aged at 20°C and food was replaced twice per week. Flies’ survival was assessed daily.

#### Behavioral assays

##### Activity monitoring

5-day-old male flies were loaded into single glass tubes (65 mm long, 5 mm external and 3 mm internal diameter) with food and housed in ethoscopes^37^ placed in a 25°C incubator with 12 h light-dark conditions. Fruit flies were followed and recorded for 6 consecutive days with a computerized video-tracking system. Videos were recorded at 2 frames per second with infrared light. The position of each animal was saved at each time point in SQLite files and subsequently analyzed with R (v 3.6.3) and rethomics with adjusted R packages behavr, scopr and sleepr (v0.3.99). The first recorded day was excluded from the analysis as it is necessary for the flies to habituate to the behavioral arena. Flies that died during the assay were also excluded from the analysis. The behavior of the monitored flies was annotated as ‘immobile’, ‘micro-movement’ or ‘walking’.

##### Seizure susceptibility assay

5-day-old flies were used for this assay. Groups of 7–10 flies were transferred to a transparent vial and startled by vortexing the vial for 10 s at maximum intensity. The behavior was scored after 10 s. Flies showing seizures or unable to walk properly -slow and uncoordinated movements-were scored as ‘impaired’. The flies were not exposed to CO_2_ on the day of the assay.

Females and males were evaluated separately. A two-way ANOVA test was performed to control for the influence of sex. As the difference between the scores of the two sexes was not different, the scores were pooled.

##### Temperature-dependent paralysis

5-day-old flies were used for this assay, and both sexes were included. Before the assay, flies were transferred into single transparent tubes and placed on a pre-heated incubator at 38°C. The time at which single flies were paralyzed -not moving for 1 min-was recorded.

#### Histology

Histological sections of fly brains were prepared^13^ by decapitating heads of aged flies (5, 15 and 25 days old) and fixing them in 4% paraformaldehyde and 2.5% glutaraldehyde in 0.1 M PB pH 7.4 overnight at 4°C or until further processing. Heads were then osmicated in 2% OsO4 for at least 2 h and subsequently incubated in 4% uranyl acetate for 1 h. After dehydration using an ethanol series, heads were embedded in hard resin (Agar 100, Laborimpex) and semi-thin (1.5 μm) sections were cut on a microtome (EM UC7, Leica) and stained on a heating block with a 1% toluidine blue (Merck) solution that includes 2% Borax for 90 s at 60°C. The stained sections were mounted with Eukit Quick-hardening mounting medium (Sigma). Histological sections were imaged using the Leica DM2500 M microscope equipped with a 40X lens and the LAS v4.0 software. ImageJ was used to quantify the vacuole area.

#### Electroretinograms (ERGs) and light-induced neurodegeneration

Light-induced neurodegeneration was induced^16^ by placing 1-3-day-old flies under continuous illumination (1300 lux) for 5 days. Then, ERGs were recorded.^87^ Flies were immobilised on glass microscope slides using double-sided tape. For the recordings, glass electrodes (borosilicate, 1.5 mm outer diameter) filled with 3 M NaCl were placed in the thorax as a reference and on the fly eye for recordings. Responses to repetitive light stimuli (1 s) given by a white light emitting diode were recorded using Axosope 10.7 and analyzed using Clampfit 10.7 software (Molecular Devices) and Igor Pro 6.37. Recordings were amplified using a Warner DP311 AC/DC amplifier (Warner Instruments) and digitized using the minidigi 1A (Molecular Devices).

#### FM1–43 dye uptake assay

Third-instar larvae were dissected in fresh Ca^2+^ free HL3, nerves were cut, and subsequently larvae were incubated for 1 min in HL3 with 4 μM FM1–43 (Invitrogen), 1.5 mM CaCl_2_ and 90 mM KCl. Multiple steps of washing with HL3 before imaging removed the non-internalized dye. Images of FM1–43 were captured with an upright widefield microscope (Nikon Eclipse FN1), fitted with 60X (NA 1.0) water dipping lens and stored using NIS elements. Mean boutonic intensities were determined, after background subtraction, using ImageJ.^88^

#### Electrophysiology

Two-electrode voltage-clamp experiments^87^ (holding potential at −60 mV) to record EJCs and mEJCs were performed in HL3 solution with 1 mM CaCl_2_. Motor nerves from muscle 6–7, segment A2 or A3 were stimulated with a suction electrode at 0.2 Hz at least 50% above the threshold. EJCs were recorded for 1 min, while mEJCs were recorded for 5 min.

Current clamp experiments^56^ to record EJPs were performed in HL3 solution with 2 mM CaCl_2_. Motor nerves from muscle 6–7, segment A2 or A3 were stimulated with a suction electrode at 10 Hz at least 50% above the threshold for 10 min. EJPs amplitudes were quantified for each of the stimuli over the 600 s stimulation duration. The amplitudes were then binned per 300 stimuli with the exception of the first 150 stimuli. The consecutive EJP amplitudes for each binned data point were normalized to the first binned data point of the first 150 stimuli.

EJCs and EJPs signals were amplified using the Axoclamp900A amplifier (Molecular Devices), filtered using a 1 kHz Bessel filter and digitized at 10 kHz using a Digidata 1440A (Molecular Devices). Data storage, processing and analysis was done using Clampfit 10.7 (Molecular Devices).

#### Transmission electron microscopy (TEM)

Third-instar larvae were dissected in cold Ca^2+^ free HL3 and immediately processed for transmission electron microscopy. Briefly, larval fillets were fixed in fresh 4% paraformaldehyde (Electron Microscopy Sciences) and 1% glutaraldehyde (Sigma) in 1 mM MgCl_2_ (Sigma) and 0.1 M Na-cacodylate (Sigma) buffer, pH 7.2, overnight at 4°C. The samples were washed with 0.1 M Na-cacodylate, pH 7.4, and osmicated with 2% osmium (OsO4/Na-Cacodylate buffer). Next, the tissue was stained with 2% uranyl acetate (Electron Microscopy Sciences) for 1.5 h and after dehydration with a grade ethanol series, samples were embedded in Agar 100 resin (Agar Scientific). Horizontal ultrathin sections (70 nm) were cut on an ultramicrotome (EM UC7, Leica) and collected on 1 × 2 mm slot, copper grids (Ted Pella, inc). Synaptic boutons were examined and imaged using a JEM-1400 transmission electron microscope (Jeol) at 80 keV. Images were quantified with ImageJ.

#### Correlative light electron microscopy (CLEM)

To correlate the evi-GFP positive structures with their ultrastructure, we resorted to CLEM.^8^^,^^16^ Third instar *dSgip1*^*−/−*^mutant larvae expressing evi-GFP in their neurons (*< nSybGal4*) were dissected in cold Ca^2+^ free HL3 and subsequently fixed for 2 h at 4°C (0.5% glutaraldehyde, 2% paraformaldehyde in 0.1 M PB, pH 7.4). After washing in 0.1 M PB, samples were stained with DAPI (Sigma). Next, near-infrared branding (NIRB) was performed using a Zeiss LSM 780 equipped with a Mai Tai HP DeepSee laser (Spectra-Physics) at 880 nm with 40% maximal power output. Z stacks of the ROI were acquired before and after branding with a 25 X water immersion lens (NA 0.8). Subsequently, after branding, samples were post-fixed (4% paraformaldehyde, 2.5% glutaraldehyde in 0.1 M phosphate buffer) overnight at 4°C. Samples were washed with 0.1 M PB and after each incubation step washed with ddH_2_O until the dehydration steps. Samples were first osmicated for 1 h (1% OsO_4_ and 1.5% potassium ferrocyanide) and then incubated in a 0.2% tannic acid for 30 min followed by a second osmication step (1% OsO_4_ for 30 min) and subsequently incubated for 20 min in 1% thiocarbohydrazide. Next, samples were osmicated for a third time (1% OsO_4_ for 30 min) and incubated overnight in 0.5% uranyl acetate. Thereafter, samples were stained with lead aspartate (Walton’s lead aspartate: 20 mM lead nitrate in 30 mM sodium aspartate, pH 5.5) for 30 min at 60°C. After a final washing step, and a dehydration series (with solutions of increasing ethanol concentration (30%, 50%, 70%, 90% and twice with 100%)), samples were twice incubated for 10 min with propylene oxide. Next, samples were infiltrated with resin agar 100 (Laborimpex), flat embedded in resin agar 100 and placed at 60°C for 48 h.

The flat resin-embedded samples were cropped into 1 mm^2^ pieces with region of interest in the middle and sectioned until the first branding marks were reached and muscle morphology was recognized by correlating with the light microscopy data. Next, ultrathin sections (70 nm) were cut on an ultramicrotome (EM UC7, Leica), collected on 1 × 2 mm slot, copper grids (Ted Pella, inc) and imaged using a JEM-1400 transmission electron microscope (Jeol) at 80 keV. NIRB branding marks around the NMJ and DAPI signal were used to correlate the confocal images with the TEM micrographs of the NMJ boutons. Overlay images were generated using ImageJ and GIMP.

### Quantification and statistical analysis

GraphPad Prism 9.3 (San Diego, USA) was used to determine statistical significance. Datasets were tested for normal distribution using the D’Agostino-Person Omnibus and Shapiro-Wilk normality tests. Normally distributed data were tested with parametric tests: when two datasets were compared, the Student’s t test was used, while when there were more than two datasets for comparison, a one-way analysis of variance test (ANOVA) followed by a post hoc Tukey test was used. For non-normally distributed datasets, Mann-Whitney test was used for bivariate comparison, and an ANOVA Kruskal-Wallis test followed by a Dunn’s post hoc test for multiple datasets. When multiple parameters were compared (genotypes and treatments) a two-way ANOVA was used, followed by a post hoc Tukey test or Šidàk test for multiple comparison correction. Significance levels are defined as ∗∗∗∗*p* < 0.0001, ∗∗∗*p* < 0.001, ∗∗*p* < 0.01, ∗*p* < 0.05 and ns, not significant. ‘n’ in the legends indicates the number of animals used and analyzed. For the confocal imaging 3–4 different NMJs were imaged in each animal. Data are plotted as mean ± SEM or SD and specifics on the statistical test used for analysis are reported in the figure legends.

## Supplemental information

Document S1. Figures S1–S4 and Tables S1–S3

Table S4. Excel listing the genotypes of the flies used in this study and the sequences of the primers, gRNA, homology arms, and gBlocks used for plasmid generation, related to STAR methods

Document S2. Article plus supplemental information
