# Supplementary material for: A candidate loss-of-function variant in SGIP1 causes synaptic dysfunction and recessive parkinsonism
Source: Cell Rep Med. 2024 Sep 26;5(10):101749. doi: 10.1016/j.xcrm.2024.101749 (PMC11513836; doi:10.1016/j.xcrm.2024.101749)
Supplement: Document S1. Figures S1–S4 and Tables S1–S3 [file mmc1.pdf]

Cell Reports Medicine, Volume *5*

Supplemental information

A candidate loss-of-function variant in SGIP1 causes synaptic dysfunction and recessive parkinsonism

Marianna Decet, Patrick Scott, Sabine Kuenen, Douja Meftah, Jef Swerts, Carles Calatayud, Sandra F. Gallego, Natalie Kaempf, Eliana Nachman, Roman Praschberger, Nils Schoovaerts, Chris C. Tang, David Eidelberg, Samir Al Adawi, Abdullah Al Asmi, Ramachandiran Nandhagopal, and Patrik Verstreken

Document S1. Supplemental data for Decet et al.

Table S1. Genetic and clinical features of patients with a biallelic mutation in the *SGIP1* gene, *related to Figure 1*.

**Characteristics**	**Subject III:1**	**Subject III:3**
Gender	Female	
Ethnicity	Arab (Omani)	
Inheritance pattern	Autosomal recessive	
Chromosome	1p31.3	
Type of mutation	Homozygous, missense	
Exon/intron	Exon 22	
cDNA change	c.2080T>G	
Protein change	p.Trp694Gly	
Protein domain	Cytoplasmic	
Parkinsonism		
Onset age (years)	19	22
Asymmetric onset	Yes	Yes
Bradykinesia	Present	Present
Rest tremor	Present	Present
Rigidity	Present	Present
Postural instability	Present	Present
Levodopa response	Present	Present
Motor fluctuations	Present	Present
Dopaminergic drug-induced dyskinesias	Mild	Mild
Dopaminergic drug-related worsening of behavioural problems	Present	Present
Hoehn-Yahr stage	4	4
Postural tremor	Absent	Present (mild)
Seizures (onset age in years)	Absent	Present (Generalised tonic-clonic seizures from age 10)
Intellectual and cognitive dysfunction	Present	Present
Supranuclear vertical gaze palsy	Absent	Absent
Pyramidal signs	Absent	Absent
Cerebellar signs	Absent	Absent
Autonomic signs	Absent	Absent
Bulbar dysfunction	Absent	Absent
Brain MRI	Normal	Normal
Brain FDG PET (Network analysis):		
PDRP	High	High
PDCP	Normal	High
MSARP	Low	Low
PSPRP	Low	Low
Automated differential diagnosis analysis (probability of PD)	99.5%	99.7%

Abbreviations: MRI= magnetic resonance imaging; MSARP= multiple system atrophy related pattern; PET= positron emission tomography; PD= Parkinson disease; PDRP= Parkinson disease-related motor pattern; PDCP= Parkinson disease-related cognitive pattern; PSPRP= progressive supranuclear palsy-related pattern.

Table S2. ROH analyses revealed five homozygous genomic regions shared between the two subjects III:1 and III:3, *related to Figure 2*.

**Chromosome**	**Start (bp)**	**End (bp)**	**Length (Mb)**
Chr1	56811604	74107645	17,3
Chr3	78657997	90485635	11,8
Chr4	108120441	114552593	6,4
Chr5	31877748	46383335	14,5
Chr20	7368303	12692140	5,3
Total			55,4

Table S3. *In silico* analysis of pathogenicity prediction of the novel *SGIP1* c.2080T>G (p.W694G) variant, *related to Figure 2*.

**Prediction Tools**	**Score (range)**	**Interpretation**
phastCons20way	0.986	Conserved
PhyloP100	7.674 (-20 to 30)	Conserved
GERP++	5.63	Uncertain
SIFT	0.001 (0 to 1)	Deleterious
PolyPhen-2	0.976 0 to 1)	Probably damaging
LRT_score	0 (0 to 1)	Deleterious
CADD	27.5	Likely deleterious
Revel	0.81 (0 to 1)	Deleterious
MUT Assesor	3 (-5.135 to 6.49)	Supporting
FATHMM	-0.45 (-16.13 to 10.64)	Uncertain
DANN	0.98 (0 to 1)	Deleterious
MetaLR	0.48 (0 to 1)	Benign
PrimateAI	0.81 (0 to 1)	Pathogenic
BayesDel	0.364 (-1.29334 to 0.75731)	Moderate Pathogenic

Figure S1

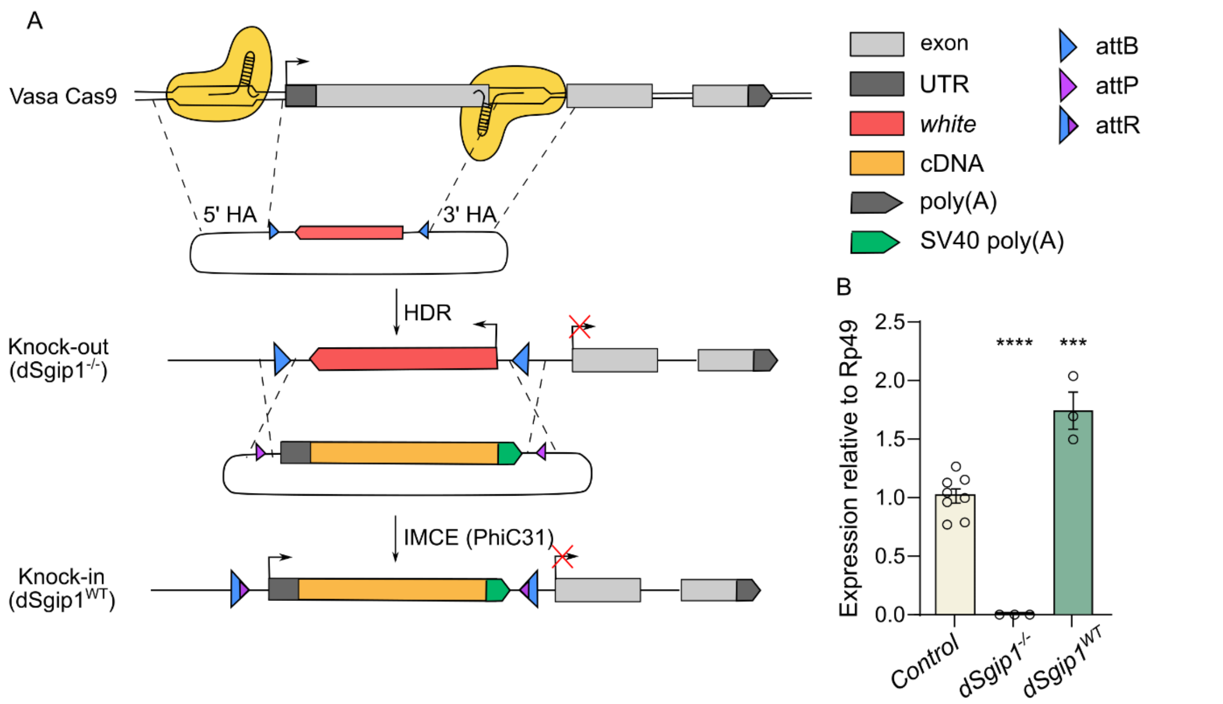

Figure S1. Two-step strategy to generate *dSgip1*$^{-/-}$ knock-out and *dSgip1*^{WT} knock-in *Drosophila*, *related to Figure 4***.**

(A) Schematic of the CRISPR/Cas9-based strategy to generate *dSgip1*$^{-/-}$ knock-out flies. In a second step, the cDNA of dSgip1^{WT} is inserted in the endogenous locus by PhiC31-mediated cassette exchange. HA: homology arm, HDR: Homology directed repair, IMCE: Integrase mediated exchange. See STAR methods. (B) Quantitative RT-PCR to assess *dSGIP1* mRNA expression levels in adult head extracts relative to Rp49. RT-PCR primers were designed against dSgip1. Statistical significance: one-way ANOVA with Dunnett's multiple comparisons test. *** $P < 0.001$, **** $P < 0.0001$. Bars: mean ± SEM, points are individual values and n≥3.

Figure S2

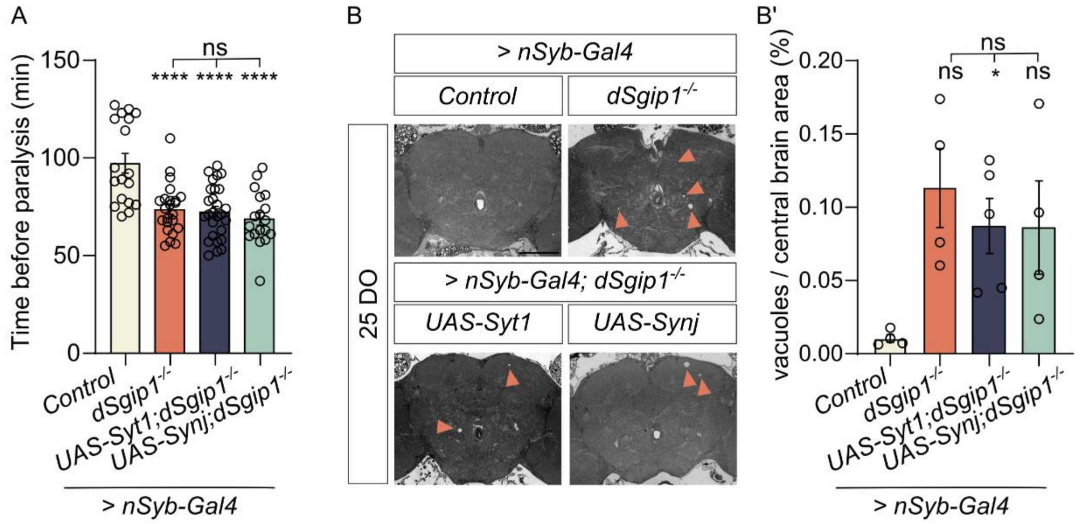

Figure S2. Overexpression of Syt1 and Synj1 do not rescue the paralysis behaviour and degeneration of *dSgip1$^{-/-}$* mutants, *related to Figure 5*.

(A) Time (min) before each fly of indicated genotypes shows complete paralysis. Flies were challenged by exposure to 38 °C. Number of tested flies ≥ 18 per genotype, two replicates. Statistical significance: one-way ANOVA. **** P < 0.0001, ns not significant, compared to control. Bars: mean ± SEM and points are individual values. (B) Widefield images of adult (25 day old) brains of flies of the indicated genotypes stained with toluidine blue. Arrowheads indicate degenerative vacuoles. Scale bar: 100 µm. (B') Quantification of the area occupied by degenerative vacuoles, expressed as percentage of central brain area. Number of analysed brains ≥ 4 per condition. Statistical significance; Brown-Forsythe and Welch ANOVA tests with a Dunnett's T3 multiple comparisons test. * P < 0.05, ns not significant, compared to control. Bars: mean ± SEM and points are individual values.

Figure S3

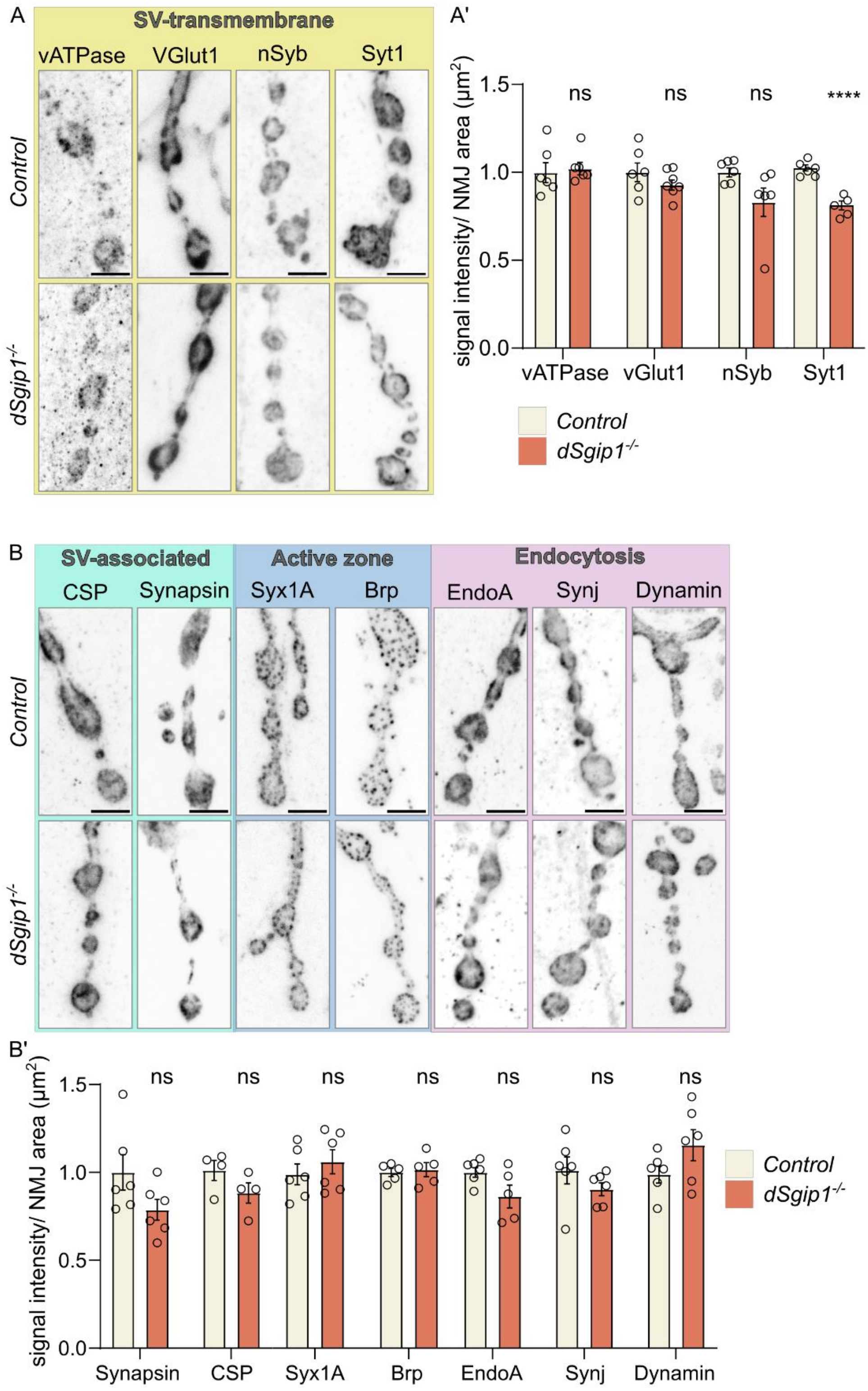

Figure S3. Synaptic protein levels in *dSgip1$^{-/-}$* mutants, *related to Figure 6***.**

(A-A') Maximum intensity projection confocal images of NMJs of control and *dSgip1$^{-/-}$* third instar larvae labelled with antibodies against the indicated SV-associated transmembrane proteins (A) and the quantification of the labelling intensity normalized to NMJ area (A'). Scale bar: 5 µm. (A'). 4 NMJs per animal were quantified, from ≥ 5 animals per condition. Statistical significance: unpaired t-test. Welch's correction applied when the variance

between the two data sets (control and *dSgip1*$^{-/-}$) was different. **** $P < 0.0001$, ns not significant. Bars: mean ± SEM and points are individual values. (B-B’) Maximum intensity projection confocal images of NMJs of control and *dSgip1*$^{-/-}$ third instar larvae labelled with antibodies against the indicated proteins (B) and the quantification of the labelling intensity levels normalized to NMJ area (B’). Scale bar: 5 µm. 4 NMJs per animal were analysed, from ≥ 5 animals per condition. Statistical significance: unpaired t-test. Welch’s correction applied when the variance between the two data sets (control and *dSgip1*$^{-/-}$) was different. ns not significant. Bars: mean ± SEM and points are individual values.

Figure S4

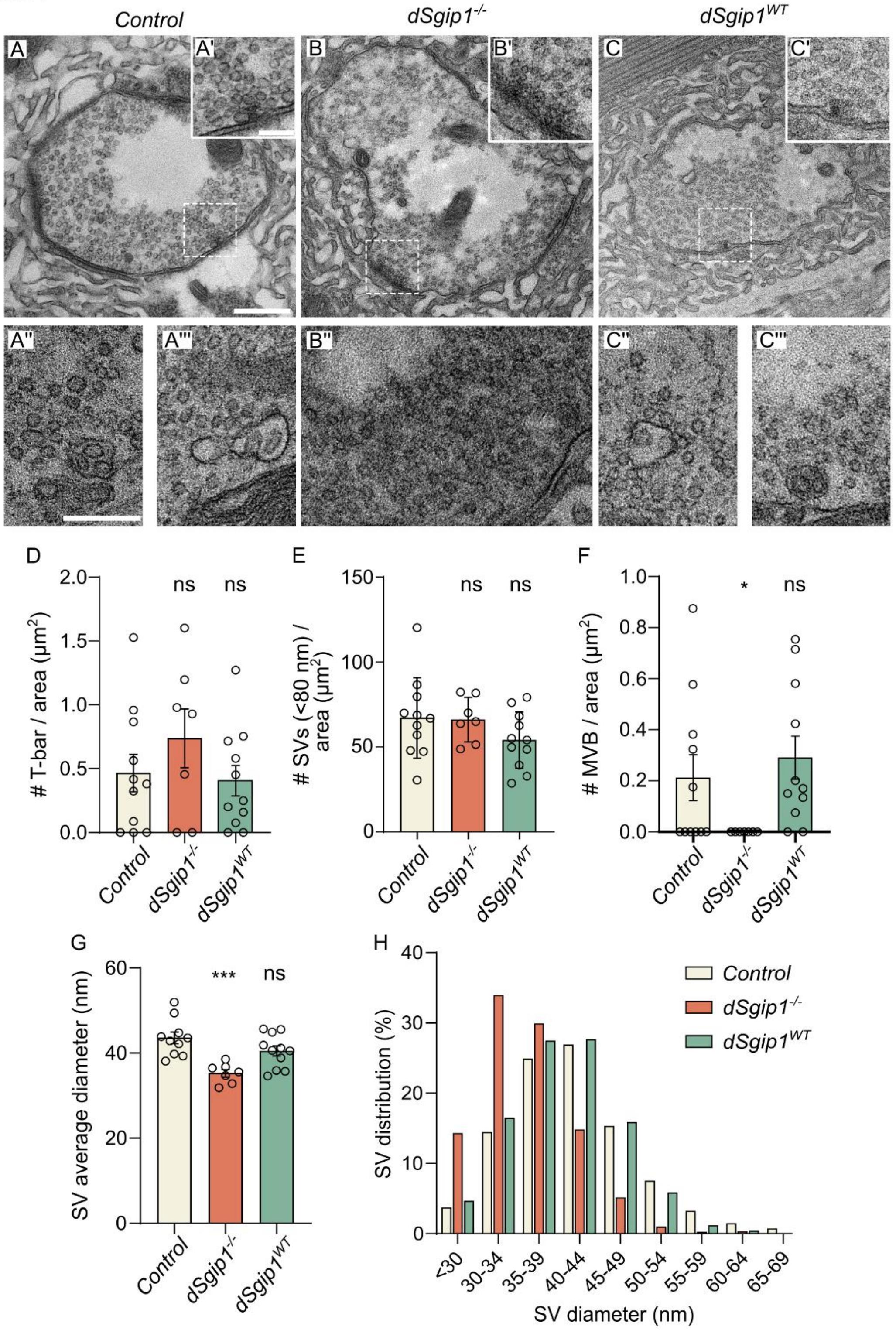

Figure S4. NMJs of *dSgip1$^{-/-}$*mutants lack multivesicular bodies, *related to Figure 7*.

(A-C) Transmission electron micrograph (TEM) images of NMJ boutons of third instar larvae of the indicated genotypes. Scale bar: 300 nm. (A'-C') Insets show enlarged areas of indicated active zone areas. Scale bar: 150 nm. (A"-C" and A'''-C''') TEM images of multivesicular bodies (MVBs) in control (A"-A''') and *dSgip1^{WT}* NMJs (C"-C''') and the lack thereof in *dSgip1$^{-/-}$* (B"). Scale bar: 250 nm. (D) Quantification of the number of active zones (T-bars) normalized to pre-synaptic bouton area of the indicated genotypes. Statistical significance: unpaired t-

test. ns not significant. Each data point represents a synaptic bouton. Number of animals ≥ 3 per genotype. Bars: mean ± SEM. (E) Quantification of the number of SVs (< 80 nm) normalized to pre-synaptic bouton area in TEM images of the indicated genotypes. Statistical significance: unpaired t-test. ns not significant. Each data point represents a synaptic bouton. Number of animals ≥ 3 per genotype. Bars: mean ± SEM. (F) Quantification of the number of multivesicular bodies (MVB) normalized to pre-synaptic bouton area in TEM images of the indicated genotypes. Statistical significance: unpaired t-test: ns not significant, * $P < 0.05$. Each data point represents a synaptic bouton. Number of animals ≥ 3 per genotype. Bars: mean ± SEM. (G) Quantification of the average diameter of SV (< 80 nm) in TEM images of the indicated genotypes. Statistical significance: unpaired t-test: ns not significant, *** $P < 0.001$. Each data point represents a synaptic bouton. Number of animals ≥ 3 per genotype. Bars: mean ± SEM. (H) Frequency distribution (in percentage) of the size of SVs (quantified in (G)) plotted in 5 nm bins.
